# Immunoproteomic identification of MbovP579, a promising diagnostic biomarker for serological detection of *Mycoplasma bovis* infection

**DOI:** 10.18632/oncotarget.9799

**Published:** 2016-06-02

**Authors:** Farhan Anwar Khan, Muhammad Faisal, Jin Chao, Kai Liu, Xi Chen, Gang Zhao, Harish Menghwar, Hui Zhang, Xifang Zhu, Muhammad Asif Rasheed, Chenfei He, Changmin Hu, Yingyu Chen, Eric Baranowski, Huanchun Chen, Aizhen Guo

**Affiliations:** ^1^ The State Key Laboratory of Agricultural Microbiology, Huazhong Agricultural University, Wuhan, China; ^2^ College of Veterinary Medicine, Huazhong Agricultural University, Wuhan, China; ^3^ Department of Animal Health, The University of Agriculture, Peshawar, Pakistan; ^4^ Key Laboratory of Development of Veterinary Diagnostic Products, Ministry of Agriculture, International Joint Research and Training Centre for Veterinary Epidemiology, Huazhong Agricultural University, Wuhan, China; ^5^ International Joint Research Centre for Veterinary Epidemiology, Huazhong Agricultural University, Wuhan, China; ^6^ INRA, UMR 1225, IHAP, Toulouse, France; ^7^ INP-ENVT, UMR 1225, IHAP, Université de Toulouse, Toulouse, France

**Keywords:** diagnostic biomarker, ELISA, immunoproteomics, immunoinformatics, Mycoplasma bovis, Immunology and Microbiology Section, Immune response, Immunity

## Abstract

A lack of knowledge regarding the antigenic properties of *Mycoplasma bovis* proteins prevents the effective control of bovine infections using immunological approaches. In this study, we detected and characterized a specific and sensitive *M. bovis* diagnostic biomarker. After *M. bovis* total proteins and membrane fractions were separated with two dimensional gel electrophoresis, proteins reacting with antiserawere detected using MALDI-TOF MS. Thirty-nine proteins were identified, 32 of which were previously unreported. Among them, immunoinformatics predicted eight antigens, encoded by Mbov_0106, 0116, 0126, 0212, 0275, 0579, 0739, and 0789, to have high immunological value. These genes were expressed in *E. coli* after mutagenesis of UGA to UGG using overlap extension PCR. A lipoprotein, MbovP579, encoded by a functionally unknown gene, was a sensitive and specific antigen for detection of antibodies in sera from both *M. bovis*-infected and vaccinated cattle. The specificity of MbovP579 was confirmed by its lack of cross-reactivity with other mycoplasmas, including *Mycoplasma agalactiae*. An iELISA based on rMbovP579 detected seroconversion 7 days post-infection (dpi). The ELISA had sensitivity of 90.2% (95% CI: 83.7%, 94.3%) and a specificity of 97.8% (95% CI: 88.7%, 99.6%) with clinical samples. Additional comparative studies showed that both diagnostic and analytic sensitivities of the ELISA were higher than those of a commercially available kit (*p*<0.01). We have thus detected and characterized the novel antigen, MbovP579, and established an rMbovP579-based ELISA as a highly sensitive and specific method for the early diagnosis of *M. bovis* infection.

## INTRODUCTION

*Mycoplasma bovis* (*M. bovis*) belongs to *Mollicutes*, a class of simple self-replicating bacteria characterized by very small genomes (580 to 2200 kb) and the lack of a cell wall. It is a major pathogen that affects cattle worldwide, causing pneumonia, mastitis, arthritis, keratoconjunctivitis, and otitis [1, 2]. In Europe, it is responsible for about a quarter of all calf pneumonia cases, causing annual losses estimated at 140 million Euros. The economic impact of *M. bovis* in the USA is similar due to mastitis and respiratory infections [1, 3]. Since 2008, *M. bovis* has been reported as a serious threat to the growing beef and dairy industry in China [4, 5].

Currently, the primary methods for controlling *M. bovis* are management practices and antimicrobial treatments [2, 3]. However, *M. bovis* is naturally resistant to antimicrobial agents targeting the cell wall, and several studies have reported low susceptibility to many commercially available antimicrobials and the emergence of resistant strains worldwide [6, 7, 8, 9, 10]. Therefore, our laboratory recently developed an effective live, attenuated vaccine for the control of *M. bovis* [3]. *In vivo* and *in vitro* studies have revealed that both virulent and avirulent strains of *M. bovis* are characterized by geno-plasticity and phenotypic diversity [4, 11]. It is therefore important to identify and characterize antigenic proteins associated with *M. bovis* infection in both virulent strains and attenuated vaccine strains to devise an effective control strategy.

Considerable efforts have been made to elucidate antigenic structures in *M. bovis*, and some relevant proteins have been identified. They include exposed surface proteins, such as variable surface proteins (Vsp), the lipoproteins P26, P48-like, and mycoplasma immunogenic lipase A (MilA), and the immunodominant membrane protein pMB67 [12, 13, 14, 15, 16, 17], as well as cytoplasmic proteins, such as heat-shock protein 60 (Hsp60), glyceraldehyde-3-phosphate dehydrogenase (GAPDH), and the pyruvate dehydrogenase beta (PDHB) subunit, among others [18, 19, 20]. The highly-conserved *M. bovis* GAPDH was suggested as a potential antigen for diagnosis or vaccines; however, a subunit vaccine based on GAPDH did not protect against *M. bovis* [21]. The cause of this poor protective efficacy is unknown, but the host Th2 response, perhaps accompanied by high levels of the weak opsonin IgG1, has been suggested [22]. In general, early diagnosis would assist in the control of *M. bovis* infection in feedlots and dairy herds. Serodiagnostic assays, which might detect the IgG specific to *M. bovis* even in chronically infected cattle or animals exposed to antimicrobial agents, may be particularly helpful in this regard [20]. Although many serodiagnostic assays have been developed [5, 17, 20], improved serodiagnostic assays based on more sensitive and specific antigens are still required for the early detection of the *M. bovis-*specific IgG in exposed cattle populations.

Because mycoplasmas lack cell walls, the cell membrane and membrane-associated proteins (MAPs) are important for mycoplasma colonization and survival within hosts. Many lipoproteins are reportedly linked to the cytoplasmic membrane, and the majority are thought to be exposed on the cell surface. However, only a few lipoproteins have been associated with virulence or antigenicity [23]. Therefore, highly conserved, sensitive, and specific antigenic membrane molecules or MAPs may be the most promising targets for establishing novel serodiagnostics.

Proteomics approaches, such as dimensional gel electrophoresis (2-DE) and MALDI-TOF mass spectrometry (MS), have increasingly been used in immunoproteomics studies of several mycoplasma species [20, 24, 25, 26, 27]. However, these data alone are often not enough to identify potential antigens containing multiple T- and B-cell epitopes [28]. Recently, immunoinformatics, a combination of immunology and informatics, has helped elucidate the networks involved in defense system activation [29]. Such methods have been used to successfully identify antigenic epitopes in several pathogens, such as *Mycobacterium tuberculosis* and *Neisseria meningitidis* [30, 31], and to select potential candidates for serodiagnostics and vaccine development [32, 33]. Analyses based on murine immunological databases may not apply to other species, including bovines. However, a combination of immunoproteomics, immunoinformatics, conventional gene expression, and subsequent immunological confirmation provides an effective method for comprehensively characterizing antigenic proteins [28].

This study was conducted to assemble a global antigenic profile for *M. bovis* using immunoproteomics and immunoinformatics and to identify promising candidate proteins in *M. bovis* using gene expression analyses and other serological methods. Among the eight identified *M. bovis* antigens expressed in *E. coli*, MbovP579 was confirmed as a conserved, sensitive, and specific antigen in both field isolates and the attenuated vaccine strain *M. bovis-*150. An indirect ELISA (iELISA) based on recombinant MbovP579 (rMbovP579) was able to effectively detect the *M. bovis-*specific IgG in animals with acute or chronic infections.

## RESULTS

### Analysis of whole cell proteins (WCPs) from *M. bovis* HB0801

**Figure 1:**
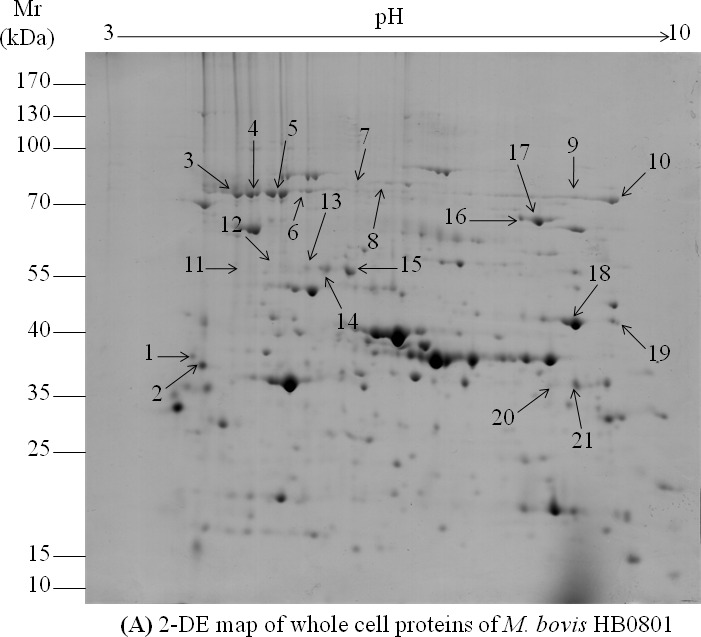

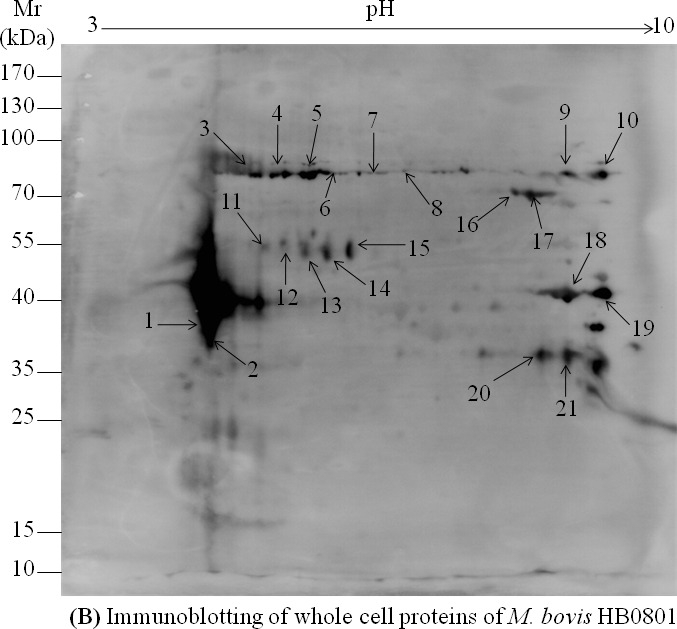
2D gel electrophoresis and western blot analysis of *M. bovis* HB0801 whole cell proteins **A.** Analysis of HB0801 whole cell proteins with 2-DE. Isoelectric points are indicated at the top and molecular weight markers in kDa on the left. **B.** Immunoblotting patterns of *M. bovis* HB0801 whole cell proteins obtained using a pool of sera derived from calves experimentally infected with HB0801. The 21 spots identified by MALDI-TOF MS are indicated on the 2D electrophoresis gel and the PVDF membrane.

Immunoproteomics revealed antigenicity of both membrane-associated and cytoplasmic proteins in the WCPs of *M. bovis* HB0801 cells. Analysis of *M. bovis* WCPs using 2-DE identified 639 well-resolved spots corresponding to 84% of the total number of coding sequences identified in the HB0801 genome (Figure 1A). Among those, 32 spots reacted with pooled sera from experimentally infected calves 35 days after infection (Figure 1B). No proteins reacted with the negative control sera. Mass spectrometry (MS raw dataset available at PRIDE repository-PXD003479) confirmed the presence of proteins in 21 spots (Figures 1A and 1B), corresponding to 16 different *M. bovis* proteins. Single spots identified 11 proteins, while five proteins were characterized by 2, 4, and 8 isoforms, suggesting post-translational modifications of these proteins (Table 1). Among these 16 antigenic proteins, 12 proteins belong to a broad range of functional categories, while 4 are hypothetical proteins with unknown functions (Table 1). Remarkably, only 7 proteins were predicted to be surface-exposed or membrane-associated: lipoproteins MbovP579 and P48-like, variable surface protein K (VspK), F_0_F_1_ ATP synthase subunit beta (AtpD), phosphonate ABC transporter substrate-binding protein, putative transmembrane protein, and a putative lipoprotein encoded by Mbov_0739 (MbovP739). The remaining nine antigenic proteins were located in the cytoplasm according to the PSORTb database (Table 1).

**Table 1:** Antigenic proteins identified in *M. bovis* HB0801 whole cell proteins

Spot no.	Gene	Protein	NCBI ID	Mw (kDa)	pI	Protein Scores	Protein Score C.I.%*	PSORTb Localization	PSORTb Probability	COG**
18	Mbov_0016	P48-like lipoprotein	gi|392051019|	51.2	8.8	816	100	Outer Membrane	9.52	R
20	Mbov_0070	Putative transmembrane protein	gi|392051070|	37.6	7.0	271	100	Cytoplasmic Membrane	10.00	-
20	Mbov_0071	Aspartate-ammonia ligase	gi|392051071|	37.8	7.1	141	100	Cytoplasmic	10.00	E
7	Mbov_0103	Pyruvate dehydrogenase beta subunit	gi|392051103|	36.1	5.4	96	99.8	Cytoplasmic	9.97	C
7-8	Mbov_0106	Dihydrolipoamide dehydrogenase (DLD)	gi|392051106|	58.9	6.3	274	100	Cytoplasmic	9.97	C
8	Mbov_0212	Transketolase (Tkt)	gi|392051206|	72.5	6.1	87	98.7	Cytoplasmic	9.97	G
14	Mbov_0304	Glycyl-tRNA synthetase	gi|392051297|	53.0	6.0	82	96.4	Cytoplasmic	10.00	J
19	Mbov_0306	Phosphonate ABC transporter substrate-binding protein	gi|392051299|	49.7	6.9	171	100	Cytoplasmic Membrane	9.97	P
7	Mbov_0481	Elongation factor Tu	gi|392051462|	43.6	5.8	64	52.5	Cytoplasmic	9.64	J
11-12-13-14	Mbov_0508	F_0_F_1_ ATP synthase subunit beta (AtpD)	gi|392051487|	50.7	5.5	273	100	Cytoplasmic Membrane	9.51	C
3-4-5-6-7-8-9-10	Mbov_0579	Lipoprotein (MbovP579)	gi|392051558|	81.6	9.0	631	100	Unknown	-	-
2	Mbov_0628	50S ribosomal protein L4	gi|392051604|	32.4	10.2	136	100	Cytoplasmic	9.26	J
16-17	Mbov_0739	Putative lipoprotein (MbovP739)	gi|392051712|	70.2	8.5	431	100	Unknown	-	-
15	Mbov_0789	Leucyl aminopeptidase (pepA)	gi|392051762|	41.0	7.0	312	100	Cytoplasmic	9.64	E
1-2	Mbov_0797	Variable surface lipoprotein K (VspK)	gi|392051770|	27.8	5.1	128	100	Unknown	-	-
21	Mbov_0839	LacI family transcriptional regulator	gi|392051809|	37.2	9.1	124	100	Cytoplasmic	9.97	K

* C.I. %: Confidence Interval

** COG (Cluster of orthologous group) database functional classes: (J) translation, ribosomal structure and biogenesis (C) energy production and conversion, (G) carbohydrate transport and metabolism, (E) amino acid transport and metabolism, (R) General function prediction only, (P) Inorganic ion transport and metabolism, (K) Transcription.

### Antigens identified *via* membrane fraction analysis of HB0801

**Figure 2:**
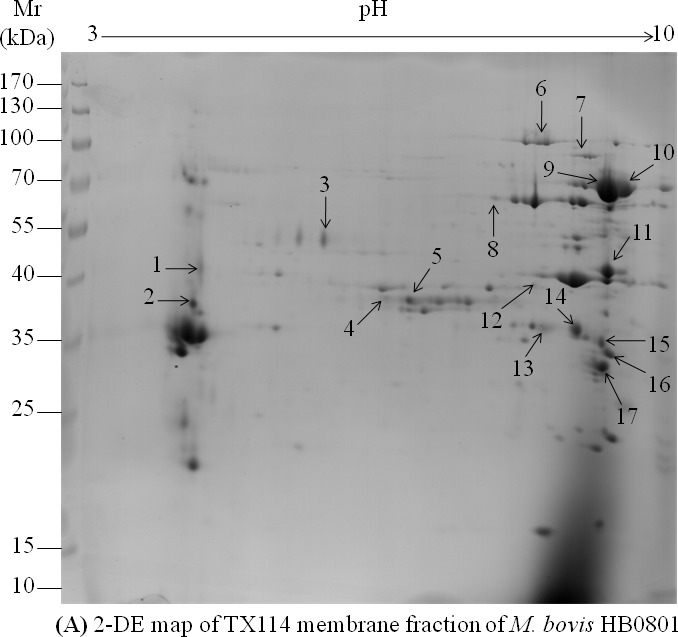

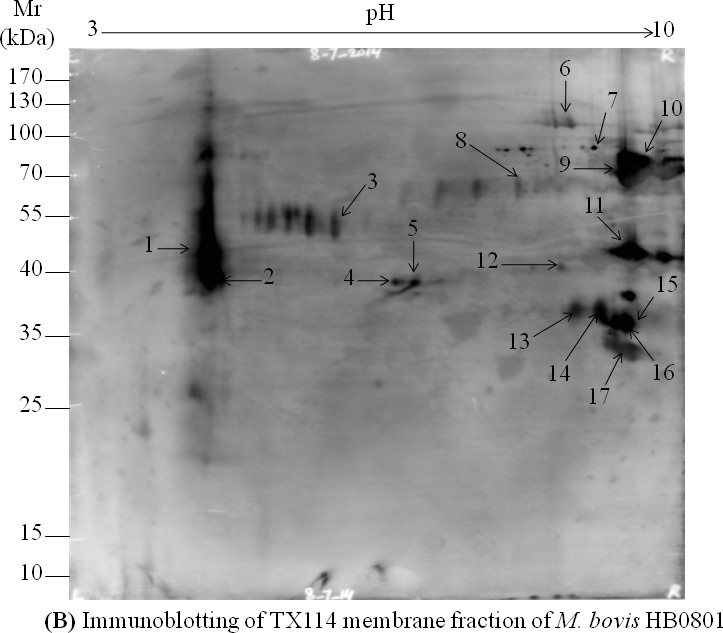
2D gel electrophoresis and western blot analysis of *M. bovis* HB0801 TX114 membrane fractions **A.** Resolution of the HB0801 TX114 membrane fraction using 2-DE. Isoelectric points, ranging from pH 3-10, are indicated at the top and molecular weight markers in kDa on the left. **B.** Antigenic pattern of the *M. bovis* HB0801 membrane fraction obtained using a serum pool from calves experimentally infected with HB0801. The 17 spots identified by MALDI-TOF MS are indicated on the 2-DE map and the PVDF membrane.

The membrane proteome of *M. bovis* was first fractionated with Triton-X-114 (TX-114) and then subjected to 2-DE to better resolve basic and liposoluble transmembrane molecules and MAPs. Most of the TX-114 soluble fractions were resolved at the basic end of a 17 cm immobilized pH gradient (IPG) strip [pH 3-10 non-linear (NL)] in the 2-DE map (Figure 2A). After Western blot analysis, protein spots that reacted strongly with antisera from experimentally infected calves 35 days after infection were excised from the 2-DE gel and subjected to MS. MS analysis (MS/MS raw dataset is available in the PRIDE repository - PXD003479) recognized the presence of proteins in 17 spots (Figure 2B), corresponding to 29 different *M. bovis* proteins. Sera from the negative control animals did not react with any of the *M. bovis* proteins. Eighteen proteins were recognized as single spots, 9 proteins were recognized in two spots, the variable surface lipoprotein [coding sequence (CDs) Mbov_0796] was identified in 4 isoforms, and cobalt/nickel ABC transporter ATP-binding protein (CDs Mbov_0594) was recognized in 3 spots (Table 2). Five of the 29 antigenic proteins were located in the cytoplasm, 10 were predicted to be MAPs, and the remaining 14 lipoproteins had unknown functions according to the PSORTb database (Table 2).

**Table 2:** Antigenic proteins in the *M. bovis* HB0801 TX114 membrane fraction

Spot no.	Gene	Protein	NCBI ID	Mw (kDa)	pI	Protein Scores	Protein Score C.I.%*	PSORTb Localization	PSORTb Probability	COG**
11	Mbov_0016	P48-like surface lipoprotein	gi|392051019|	51.2	8.8	138	100	Outer Membrane	9.52	R
17	Mbov_0038	putative transmembrane protein	gi|392051041|	379.67	7.9	76	99.534	Outer Membrane	9.49	-
6-9	Mbov_0111	putative lipoprotein	gi|392051111|	110.11	8.45	1,080	100	Unknown	-	-
9	Mbov_0115	Oligopeptide ABC transporter ATP-binding protein"	gi|392051115|	94.0	8.9	57	62.107	Cytoplasmic Membrane	9.99	E
17	Mbov_0116	putative lipoprotein (MbovP116)	gi|392051116|	37.4	9.1	60	81.441	Unknown	-	-
4-5	Mbov_0126	XAA-Pro aminopeptidase (pepP)	gi|392051126|	39.8	6.05	460	100	Cytoplasmic	9.97	E
16-17	Mbov_0130	putative transmembrane protein	gi|392051130|	26.3	9	64	91.319	Unknown	-	S
15	Mbov_0156	putative lipoprotein	gi|392051154|	37.1	8.85	201	100	Unknown	-	-
11	Mbov_0217	putative lipoprotein	gi|392051211|	53.5	9.13	342	100	Unknown	-	-
13	Mbov_0275	putative membrane lipoprotein (MbovP275)	gi|392051268|	40.1	8.51	118	100	Unknown	-	-
1	Mbov_0283	Putative lipoprotein	gi|392051276|	28.9	5.14	79	99.7	Unknown	-	-
3	Mbov_0299	NADH oxidase	gi|392051292|	49.9	6.09	76	99.4	Cytoplasmic	9.97	R
11	Mbov_0306	Phosphonate ABC transporter substrate-binding protein	gi|392051299|	51.5	9.04	374	100	Cytoplasmic Membrane	9.97	P
4-5	Mbov_0312	Alcohol dehydrogenase	gi|392051305|	37.9	6.51	284	100	Cytoplasmic	9.97	ER
12	Mbov_0468	putative lipoprotein	gi|392051449|	65.5	8.35	255	100	Unknown	-	-
7	Mbov_0505	putative lipoprotein	gi|392051486|	90.4	7.64	411	100	Unknown	-	-
17	Mbov_0512	hypothetical protein	gi|392051491|	36.4	8.93	62	85.921	Cytoplasmic	8.96	-
9	Mbov_0515	Putative lipoprotein	gi|392051494|	97.5	8.81	58	71.255	Outer Membrane	9.52	-
7-9	Mbov_0517	Putative transmembrane protein	gi|392051496|	83.9	8.34	168	100	Outer Membrane	9.49	-
10-17	Mbov_0579	lipoprotein (MbovP579)	gi|392051558|	81.6	9.1	980	100	Unknown	-	-
12	Mbov_0580	Nuclease	gi|392051559|	32.9	6.92	154	100	Extracellular	9.97	L
13-14-15	Mbov_0594	cobalt/nickel ABC transporter ATP-binding protein	gi|392051573|	35.3	6.69	155	100	Cytoplasmic Membrane	8.78	P
16-17	Mbov_0693	putative transmembrane protein	gi|392051667|	302.21	8.41	70	97.819	Extracellular	8.91	-
7-8	Mbov_0739	Putative lipoprotein (MbovP739)	gi|392051712|	70.2	8.45	214	100	Unknown	-	-
3	Mbov_0789	Leucyl aminopeptidase (pepA)	gi|392051762|	50.4	5.63	84	99.9	Cytoplasmic	9.64	E
13-14-15-16	Mbov_0796	variable surface lipoprotein	gi|392051769|	31.5	9.01	350	100	Unknown	-	
2	Mbov_0797	Variable surface lipoprotein	gi|392051770|	34.6	5.02	324	100	Unknown	-	-
15-16	Mbov_0798	variable surface lipoprotein	gi|392051771|	39.8	9.06	105	100	Unknown	-	-
3	Mbov_0838	Putative lipoprotein	gi|392051808|	50.3	6.95	133	100	Periplasmic	9.83	-

* C.I. %: Confidence Interval

** COG (Cluster of orthologous group) database functional classes: (E) amino acid transport and metabolism, (R) General function prediction only, (P) Inorganic ion transport and metabolism, (L) Replication, recombination and repair

### Animal infection and immunization

Increased titers of serum antibodies and gross lesion evaluation in postmortem examinations confirmed the success of animal infection experiments. Antibody levels were increased in the infected and immunized calves compared to negative control animals. Different lesions typical of infection, such as consolidation at the apical lung lobe and adhesions between the chest wall and the lung surface, were only observed in the infected group, and the mean pathological score of infected calves (22.7±5.1) was higher than that of immunized (5.3±3.1) and control (3.3±1.5) calves *(p* < 0.01).

### Immunoblotting of recombinant membrane lipoproteins and cytoplasmic proteins

**Figure 3:**
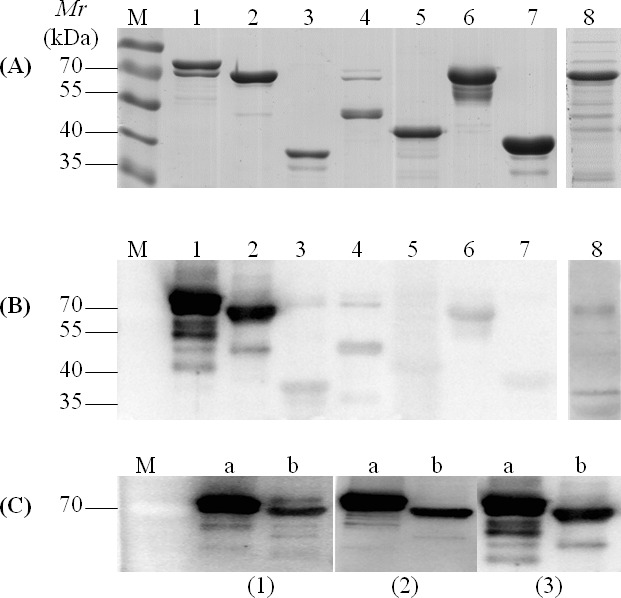
Representative SDS-PAGE and Western blotting assay of recombinant proteins **A.** SDS-PAGE of recombinant proteins; **B.** Immune-reactivity of all recombinant proteins with antiserum samples from naturally infected cattle; **C.** Immunoblotting of recombinant lipoproteins (rMbovP579 and rMbovP739) with antisera from experimental calves 35 dpi (1), experimental calves 35 days post-vaccination (2), and naturally infected cattle (3). Protein markers are indicated by “M” with the respective molecular weights in kDa on the left. Lanes 1-8 in **A.** and **B.** indicate proteins rMbovP579, rMbovP739, rMbovP116, rpepA, rpepP, rDLD, rMbovP275, and rTkt, respectively; lanes **A.** and **B.** in **C.** indicate rMbovP579 and rMbovP739, respectively. Serum samples from each group of cattle were diluted at 1:100.

The antigenic proteins identified by immunoproteiomics were analyzed *in silico* to select candidate proteins for further evaluation. Based on immunoinformatics analysis, 8 proteins (Table 3) were predicted to have a large number of T- and B-cell epitopes (Table S7-S10). These 8 highly-conserved antigenic *M. bovis* proteins (Table 3) were cloned and expressed in *E. coli*. The genes were first subjected to a site-directed mutagenesis (UGA to UGG) using overlapping PCR. Collectively, 25 site mutations were performed in the 8 genes (Table S1). The full genes were then cloned into pET-30a (+) vectors and sequenced. Alignment assays confirmed the success of mutagenesis; the cloned genes matched the published sequence of HB0801 at the nucleotide level, except for the above modifications, and were inserted into the vector correctly. Expression of recombinant proteins was then induced with isopropyl-b-D-1-thiogalactopyranoside (IPTG, Invitrogen) and confirmed with SDS-PAGE (Figure 3A). After purification based on the his-tag, the 8 recombinant proteins were separated by SDS-PAGE, transferred to a membrane, and probed with antisera from both experimentally and naturally infected animals. rMbovP579 and recombinant MbovP739 (rMbovP739) were identified using serum antibodies from naturally infected animals, sera from calves 35 days after experimental infection with HB0801, and serum samples from calves immunized with *M. bovis*-150 35 days after immunization (Figure 3C). The other 2 lipoproteins and all 4 recombinant cytoplasmic proteins (Table 3) displayed weak signals in response to sera from all animal groups (Figure 3B).

**Table 3:** List of *M. bovis* HB0801 antigens expressed in *E. coli*

Antigenic proteins of M.bovis HB0801 with NCBI protein ID	Mnemonic in *M.bovis* HB0801	Homologs of HB0801 antigens in other sequenced *M. bovis* strains								
		*M. bovis* PG45			*M. bovis* Hubei-1			*M. bovis* CQ-W70		
		Gene name	Identity %	NCBI protein ID	Gene name	Identity %	NCBI protein ID	Gene name	Identity %	NCBI protein ID
Putative lipoprotein^a^ (MbovP116) (AFM51491.1)	Mbov_0116	MBOVPG45_0117	94	ADR25135.1	MMB_0110	100	AEI89824.1	K668_00555	100	AIA33703.1
Putative membrane lipoprotein^a^ (MbovP275) (AFM51643.1)	Mbov_0275	MBOVPG45_0584	96	ADR24848.1	MMB_0254	100	AEI89968.1	K668_01285	100	AIA33843.1
Lipoprotein (MbovP579)^a^ (AFM51933.1)	Mbov_0579	MBOVPG45_0311	92	ADR25025.1	MMB_0540	100	AEI90254.1	K668_02710	100	AIA34118.1
Putative lipoprotein (MbovP739)^a^ (AFM52087.1)	Mbov_0739	MBOVPG45_0747	92	ADR24729.1	MMB_0704	100	AEI90416.1	K668_03510	100	AIA34272.1
Dihydrolipoamide dehydrogenase (DLD)^b^ (AFM51481.1)	Mbov_0106	MBOVPG45_0108	100	ADR24676.1	MMB_0100	100	AEI89814.1	K668_00505	100	AIA33693.1
XAA-Pro aminopeptidase (pepP)^b^ (AFM51501.1)	Mbov_0126	MBOVPG45_0127	99	ADR24864.1	MMB_0120	100	AEI89834.1	K668_00605	100	AIA33713.1
Transketolase (tkt)^b^ (AFM51581.1)	Mbov_0212	MBOVPG45_0650	99	ADR25127.1	MMB_0198	100	AEI89912.1	K668_01000	100	AIA33788.1
Leucyl aminopeptidase (pepA)^b^ (AFM52137.1)	Mbov_0789	MBOVPG45_0802	100	ADR25327.1	MMB_0755	100	AEI90467.1	K668_03765	100	AIA34323.1

a Membrane-associated protein

b Cytoplasmic protein

### Characterization of the antigenicity of recombinant proteins with iELISA

**Figure 4:**
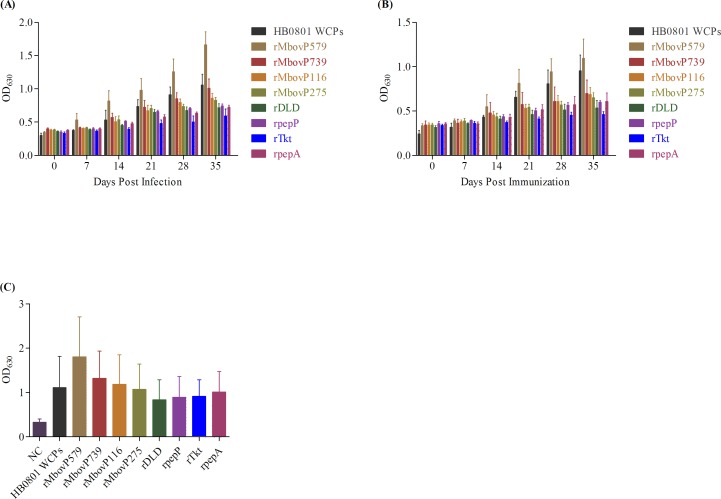
Reactions of *M. bovis* HB0801 whole cell proteins and recombinant proteins to different antisera measured with iELISA *M. bovis* HB0801 whole cell proteins (WCPs) and all recombinant proteins (rMbovP579, rMbovP739, rMbovP116, rMbovP275, rDLD, rpepP, rTkt, and rpepA) were characterized *in vivo* with antiserum samples from each experimentally infected calf 0-35 dpi **A.**, antisera from each experimentally immunized calf 0-35 days post-vaccination **B.**, and serum samples from each animal naturally infected with *M. bovis* **C.**. The Y-axis indicates mean ± SD iELISA OD_630_ values of serum antibodies from infected, vaccinated, and uninfected (NC) animals against WCPs and all recombinant proteins. Serum antibodies to proteins were diluted as follows: WCPs 1:400, rMbovP579 1:1600, rMbovP739 1:200, and rMbovP116, rMbovP275, rDLD, rpepP, rTkt, and rpepA 1:100.

The *in vivo* expression and antigenicity of serum antibodies for *M. bovis* HB0801 WCPs and all 8 recombinant proteins (Table 3) were analyzed using antisera from both experimentally and naturally infected animals. In an iELISA based on the *M. bovis* WCPs, 40% (8/20) of experimentally infected calves 14 days post-infection (dpi) and all infected calves 21 dpi were positive for antibody responses, whereas none of the vaccinated calves were positive until 21 days after immunization (Figures 4A and 4B). As expected, no antibody response against *M. bovis* WCPs was detected in the uninfected animals at any time point or in calves before infection or on the day of vaccination.

In contrast to the immunoblotting assays, iELISA demonstrated that all of the recombinant proteins generated antibody responses in both experimentally and naturally infected animals, indicating that recombinant protein antigenicity depended on their 3-dimensional structures in addition to primary structures.

Based on the intensity of antibody responses, the 4 recombinant membrane-associated lipoproteins (Table 3) together induced higher antibody responses than the 4 recombinant cytoplasmic proteins (recombinant dihydrolipoamide dehydrogenase (rDLD), XAA-Pro aminopeptidase (rpepP), transketolase (rTkt), and leucyl aminopeptidase (rpepA)). Remarkably, the IgG response to rMbovP579 was highest (*p <* 0.001), and rMbovP739 elicited the second-highest response (*p <* 0.05), in all animal groups compared to the other recombinant membrane lipoproteins and cytoplasmic proteins.

Serum antibodies were detected in 5% (1/20) of calves 7 dpi and in all infected animals 14 dpi in the rMbovP579-based iELISA. While sera from 62.5% (5/8) of the vaccinated animals showed positive antibody responses in the rMbovP579-based iELISA 14 days after immunization, sera from all vaccinated animals showed positive responses after 35 days (Figures 4A and 4B). In the rMbovP739 assay, sera from 30% (6/20), 55% (11/20), and 100% of infected calves were positive 14, 21, and 35 dpi, respectively (Figures 4A and 4B), whereas antibody levels in immunized animals were lower than those in infected calves throughout the experiment, likely due to the lower dose administered to vaccinated animals. In assays based on recombinant lipoproteins encoded by CDs Mbov_0116 (rMbovP116) and CDs Mbov_0275 (rMbovP275), sera from 20% (4/20) of the infected calves and 25% (2/8) of the vaccinated calves were positive 14 days after treatment; sera from all calves were positive by day 35 post-infection/vaccination, although antibody levels were relatively low (Figures 4A and 4B). Antisera responses to rDLD, rpepP, rTkt, and rpepA were low compared to responses to lipoproteins for both experimentally infected and immunized calves (Figures 4A and 4B).

Among the recombinant lipoproteins, titers of antibodies against rMbovP579 and rMbovP739 were higher than those against the cytoplasmic proteins rDLD, rpepP, rTkt, and rpepA in the sera from naturally infected animals (*p* < 0.01). Meanwhile, the reactivity of rDLD, rpepP, rTkt, and rpepA with antisera was higher for naturally infected animals than for experimentally infected animals (Figure 4C). Additionally, the reaction of rMbovP579 with serum antibodies in naturally infected animals was higher than that of the other lipoproteins (*p* < 0.01) (Figure 4C).

### Specificity of the highly antigenic lipoproteins

**Figure 5:**
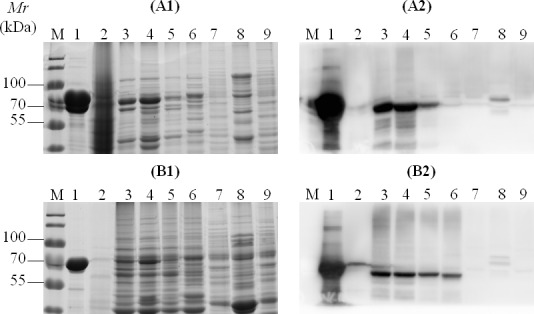
MbovP579 and MbovP739 expression in *M. bovis* strains and other mycoplasmas **A1.** SDS-PAGE of rMbovP579 (81.6 kDa) and WCPs of mycoplasmas; **A2.** Western blot analysis with mouse anti-rMbovP579 polyclonal antibodies; **B1.** SDS-PAGE of rMbovP739 (70.2 kDa) and WCPs of mycoplasma strains; **B2.** Western blot analysis with mouse anti-rMbovP739 polyclonal antibodies. Protein markers are indicated by “M” with the respective molecular weights in kDa on the left. Lanes 1-9: rMbovP579/rMbovP739, *E. coli* (BL 21-DE_3_) WCPs as a negative control, HB0801, M. bovis-150, PG45, PG2, PG3, Y98, and *M. arginini*, respectively. Serum samples were diluted at 1:100.

The highly antigenic lipoproteins (MbovP579 and MbovP739) were analyzed further. Polyclonal antibodies against rMbovP579 and rMbovP739 were raised in Balb/c mice to determine cross-reactivity with other common ruminant pathogens. Western blot analysis with these monospecific antibodies was used to identify the expression of these proteins in the *M. bovis* HB0801, attenuated *M. bovis*-150, and *M. bovis* typed (PG45) strains. No cross-reactivity was observed with WCPs from *E.coli* (BL21-DE_3_ strain) or other mycoplasma species, including *Mycoplasma mycoides* subsp. *capri* (PG3), *Mycoplasma ovipneumoniae* (Y98), and *Mycoplasma arginini*. Moreover, while the rMbovP739 antibody displayed cross-reactivity with *Mycoplasma agalactiae* (PG2) WCPs, no cross-reactivity was observed between the rMbovP579 antibody and other mycoplasma WCPs, demonstrating its high specificity for *M. bovis* (Figures 5A.2 and 5B.2). Amino acid sequence homologies for MbovP579 with other mycoplasmas are shown in Table 4. No IgGs reactive to both rMbovP579 and rMbovP739 were identified in reference antiserum against IBRV and BVDV with a WCPs-based iELISA.

**Table 4:** Homologues of *M. bovis* HB0801 lipoprotein (MbovP579) in other mycoplasmas

Pathogen	Mnemonics	Predicted gene function	Query coverage (% of residue)	Identity %
*M. bovis* PG45	MBOVPG45_0311	Membrane lipoprotein P81	98	92
*M. bovis* Hubei-1	MMB_0540	P80, lipoprotein	100	100
*M. bovis* CQ-W70	K668_02710	Membrane lipoprotein P81	100	100
*M.bovis* NM 2012	AAV31_02845	Lipoprotein	100	99
*M. agalactiae* PG2	MAG5030	P80, predicted lipoprotein	99	66
*M. agalactiae* 14628	MAGb_1420	P80, predicted lipoprotein	100	66
*M. mycoides* subsp. *mycoides* SC str. PG1	MSC_0519	Prolipoprotein B	52	22
*M. bovigenitalium* 51080	MBVG_0790	Hypothetical protein	97	27
*M. mycoides* subsp. *capri* PG3	MMC_3250	Hypothetical protein, predicted lipoprotein	10	25
*M. mycoides* subsp. *capri* GM12	MMCAP2_0459	Glycerol ABC transporter	52	23
*M. capricolum* subsp. *capricolum* ATCC 27343	MCAP_0451	Putative lipoprotein	10	28
*M. ovipneumoniae* Y98	-	-	-	No similarity
*M. ovipneumoniae* 14811	MOVI_3720	Hypothetical protein	88	24
*M. arginini* ATCC 23838	-	-	-	No similarity
*M. arginini* 7264	MARG_2010	Hypothetical protein	97	24
*Mycoplasma hominis* ATCC 27545	MLBD4_00370	Putative polynucleotide-binding lipoprotein	96	25

### Performance of the rMbovP579-based iELISA

**Figure 6:**
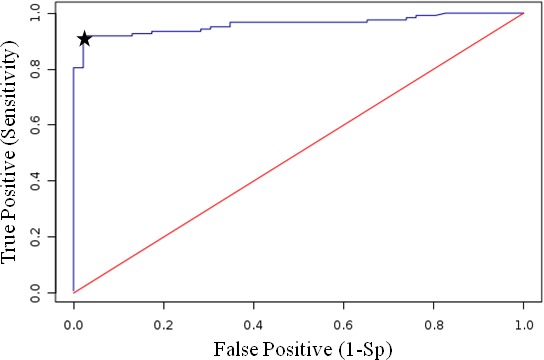
ROC analysis of the rMbovP579-based iELISA compared to gold standard tests Based on the ROC curve, 0.442 was determined as the iELISA cut-off value with a sensitivity of 90.2% and specificity of 97.8%.

Because of the specificity of MbovP579 and its conservation in all sequenced *M. bovis* strains (Table 3), including all other sequenced Chinese strains (unpublished data) (Table S2), an iELISA based on rMbovP579 was developed to confirm its value as a diagnostic biomarker. Optimal concentrations of the antigen and positive and negative sera were determined separately for the iELISA. The OD_630_ ratios of positive (P) to negative sera (N) were analysed. Based on a high P/N ratio and OD_630_ values of approximately 1.00 for positive serum samples, the optimal antigen and serum dilutions were determined to be 250 ng/well and 1:1600, respectively, with a P/N = 2.293. The optimal secondary antibody dilution was 1:5000 (v/v), and the cut-off value (OD_630_) was calculated as 0.442 by ROC analysis (Table S3). Compared to the gold standard test (*M. bovis* isolation and characterization), the diagnostic sensitivity of the rMbovP579-based iELISA was 90.2% (95% CI: 83.7%, 94.3%) and the specificity was 97.8% (95% CI: 88.7%, 99.6%) for sera from naturally infected and uninfected control animals (Figure 6 and Table S3, S4). For sera from experimentally infected and negative control calves, diagnostic sensitivity was 100% (20/20) and specificity was 100% (8/8) (Table S5). The estimated Kappa agreement value between the two methods was 0.7108. Furthermore, the specificity of this iELISA was determined by testing the antisera against common cattle pathogens, including infectious bovine rhinotracheitis virus (IBRV) and bovine viral diarrhoea virus (BVDV). OD_630_ values were less than 0.40 (0.207±0.042) for all serum samples, confirming the specificity of this iELISA.

### Comparison of efficiency between rMovP579-based iELISA and a commercial kit

Parallel serum samples from 20 experimentally infected calves and 123 naturally infected cattle were tested using the rMbovP579-based iELISA and a commercially available kit. All experimentally infected animals (20/20) were identified as positive with the rMbovP579-based iELISA, whereas only 65% (13/20) were positive according to the commercial kit. In addition, the sensitivity of the iELISA for these samples was 90.2% (95% CI: 83.7%, 94.3%) compared to only 31.7% for the commercial kit. The serum samples identified as negative for the *M. bovis*-specific IgG by the iELISA were also found to be negative with the commercial kit (Table S6). These results demonstrate that the rMbovP579-based iELISA was more sensitive than the commercial kit (*p* < 0.01). To determine the analytic sensitivity of both assays, 93 positive and 25 negative sera were individually two-fold serially diluted (1:100 - 1:102,400) and evaluated separately using both methods. The iELISA detected the IgG at dilutions as high as 1:51,200; however, the maximum detectable dilution for the commercial kit was 1:6400 with an 8-fold decrease. Thus, the analytic sensitivity of the rMbovP579-based iELISA was higher than that of the commercial kit (*p* < 0.05).

### Unique characteristics of MbovP579

The specificity of MbovP579 was further confirmed by a gene (Mbov_0579) sequence assay. Alignments of the *M. bovis* HB0801 Mbov_0579 gene with other sequenced *M. bovis* strains, including PG45 (CP002188.1), Hubei-1 (CP002513.1), CQ-W70 (CP005933.1), and NM2012 (CP011348.1), showed 95%, 100%, 100%, 99% gene-level similarities, respectively, while the nucleotide sequence was 74% similar to a homologous gene in *Mycoplama agalactiae*. The lack of genes orthologous to Mbov_0579 in other pathogens further supported its specificity as a diagnostic antigen.

Using SignalP4.1 and PRED-LIPO, MbovP579 was identified as a secreted peptide with reliability scores of 0.591 and 0.96, respectively. The MbovP579 signal peptide sequence, MSKKNKLMIGLSSTAIPLLAAVSA|KC, was a 24-amino-acid fragment at the N-terminal and contained a putative peptidase I cleavage site between the alanine and lysine residues at positions 24 and 25, resulting in the release of a mature protein with lysine as the first residue. Interestingly, MbovP579 secretion was also predicted by a nonclassical secretory pathway with a high SecP score of 0.84.

In addition, the predicted template structure for MbovP579 according to ElliPro was an ADP-ribosyltransferase toxin known as *Clostridium difficile* toxin subunit “a” (CDTa) (PDB ID- 2WN4) (Figure S-1A). CDTa is believed to have catalytic activity. Alignment of the MbovP579 and CDTa amino acid sequences revealed similarity of approximately 31% for amino acids residues 591-674 at the MbovP579 C-terminus (Figure S-1B).

## DISCUSSION

*Mycoplasma bovis* infection is a major threat to the worldwide cattle industry. It was initially detected in China in 2008 as an emerging pathogen in feedlots and continues to cause economic losses. Due to the lack of an efficient preventive strategy, the control of *M. bovis* is largely reliant on management practices and early chemotherapy. Therefore, researchers have focused on diagnostic laboratory tests for the early detection and subsequent treatment of diseases related to *M. bovis* infection in beef and dairy herds. Generally, serological assays are highly reliable because antibodies remain detectable for several months in infected animals, even after antibiotic treatments [20, 23]. Additionally, because antigenic variation is prevalent in both virulent and attenuated *M. bovis* strains [4, 11], more conserved, sensitive, and specific antigens for *M. bovis* are urgently required to help develop reliable serodiagnostic methods for the early detection of infection and assessments of vaccine efficacy. Here, we identified several previously unreported antigenic *M. bovis* proteins and characterized a promising candidate diagnostic biomarker protein.

### Global antigenic profile of *M. bovis* identified using immunoproteomics

During the last decade, immunoproteomics approaches, such as 2-DE combined with Western blotting and MS, have increasingly been used to detect antigens in many pathogens, including several mycoplasma species [20, 24, 25, 26, 34, 35]. In this study, we analyzed *M. bovis* WCPs together with membrane fractions using immunoproteomics for the first time to comprehensively identify antigenic *M. bovis* proteins. Collectively, 39 antigenic proteins were identified, 6 of which were present in both the WCPs and membrane fractions. Thirty-two novel antigenic proteins, most of which were MAPs, were identified (Table 1, 2). The discovery of 23 additional antigens in the *M. bovis* membrane fractions confirmed the importance of membrane proteins in pathogen antigenicity.

### Confirmation of previously identified antigens

Five of the antigenic proteins in our protein list, VspK, PDHB, DLD, P48-like lipoprotein, and MilA (encoded by CDS Mbov_0693 in HB0801), have previously been identified in other *M. bovis* strains. In addition, the antigenicity of DLD has been reported in many other ruminant mycoplasma species, including several members of the mycoides phylogenetic cluster [20, 24, 25, 35].

Three other important antigenic proteins in HB0801 identified here are AtpD, TktA, and the elongation factor Tu (Ef-Tu). Although they have been identified as antigens in other mycoplasma species, these proteins have not been identified in previous studies of *M. bovis*. AtpD has been used in a serological assay for the diagnosis of *M. pneumonia* infections in humans [36]. AtpD immunoreactivity has also been reported in *M. mycoides* subsp*. mycoides* SC, *Brucella abortus,* and *Brucella melitensis* [24, 37, 38]. Similarly, the antigenic properties of TktA and Ef-Tu have been reported in several mycoplasma species [24, 25, 35, 39]. However, AtpD might have lower specificity as a diagnostic marker for *M. bovis* due to its 83% similarity with the amino acid sequence of AtpD in *M. mycoides* subsp*. mycoides* SC and *M. mycoides* subsp*. capri* (NCBI database).

Three of the proteins we identified, P48-like lipoprotein, MilA, and PDHB, have been evaluated for serodiagnosis of *M. bovis* [5, 17, 20]; however, assays based on P48 and rPDHB have relatively low sensitivities of 56.3% and 51%, respectively, in sera from naturally infected animals. The sensitivity and specificity of the recombinant MilA-based assay for clinical bovine serum samples have not yet been determined [17].

*Vsp*K is a phase variable antigen encoded by a cluster of hypervariable surface exposed lipoproteins, called the *Vsp* locus, in *M. bovis* [15]. The antigenic- and virulence-related properties of *Vsp*s and pMB67 (a *Vsp*-unrelated variable membrane protein) have been described elsewhere [12, 13]. Although *Vsp*K exhibited a strong reactivity with animal sera, immune response to *Vsp*s is non-protective due to variations in expression, conformation, and antigenicity [17, 40]. Thus, highly variable antigenic proteins, such as *Vsp*K and pMB67, are not the best options for serodiagnostic assays or subunit vaccine development.

Other *M. bovis* antigens identified in previous studies, such as pMB67, heat shock protein 60, and GAPDH, were not detected in this study. This might be due to differences in methods and antisera used for antigen identification. For instance, pMB67 was detected to be antigenic in *M. bovis* total proteins by radiolabeling, SDS-PAGE, and immunostaining using monoclonal antibodies [12], while the antigenicity of recombinant Hsp60 [18] and GAPDH [19] were identified by conventional serological methods with antisera from naturally infected animals. Here, proteomic technology and antisera from experimentally infected animals were used to identify antigenic proteins, which were then confirmed using antisera from naturally infected animals. It is possible that these two types of antisera may identify different antigenic profiles for *M. bovis* strains due to differential infection doses and courses in experimental *versus* natural infection.

### The novel rMbovP579-based iELISA is the most sensitive and specific method for detecting *M. bovis*-specific antibodies

Among the 8 antigenic recombinant proteins discovered in this study, MbovP579 was the most sensitive, specific, and conserved antigen. An rMbovP579-based iELISA was thus established. This iELISA could detect seroconversion 7 dpi and by day 14 after vaccination. To the best of our best knowledge, this iELISA, which was far more sensitive than a commercially available kit, may currently be the most sensitive and specific method for *M. bovis* antigen detection.

Previous assays often failed to differentiate antibody responses against *M. bovis* from those against *M. agalactiae* infection because these mycoplasma species are closely phylogenetically related. Surprisingly, the rMbovP579-based iELISA was able to differentiate the antibody responses induced by these two species. Nucleotide alignment revealed that the Mbov_0579 gene is not only conserved among *M. bovis* strains, but is also unique to *M. bovis* compared to other pathogens. For example, the similarity of this gene at the nucleotide level is only 74% between *M. bovis* and *M. agalactiae*, and no orthologues were found in other pathogens.

According to published data [4], the function of the MbovP579 lipoprotein is unknown. We performed a bioinformatics assay to further characterize this protein. It was predicted to be a secretory lipoprotein since it has a 24-amino-acid signal sequence at the N-terminal [41, 42, 43]. More interestingly, the predicted MbovP579 template structure was ADP- ribosyltransferase CDTa (PDB ID- 2WN4). The functional domains of ADP-ribosyltransferase have recently been identified in the community-acquired respiratory distress syndrome (CARDS) toxin of *Mycoplasma pneumonia* [44]. In this study, using the ElliPro modeling tool, we predicted that MbovP579 might be an ADP-ribosyltransferase toxin, although the amino acid similarity between the MbovP579 C-terminus from amino acids 591-674 and CDTa is only 31%. Whether it functions as protein toxin remains to be established in future studies.

In conclusion, we identified and characterized MbovP579 as a promising novel diagnostic biomarker for *M. bovis* using immunoproteomics. The highly sensitive and specific rMbovP579-based iELISA would likely aid in the early detection of *M. bovis* infection.

## MATERIALS AND METHODS

### Ethical statement about animal experiments

Experimental protocols involving animals were approved by the Hubei Province Science and Technology Department, which is responsible for ethics in animal experiments in Hubei, China (permit no. SYXK(ER) 2010-0029), in accordance with China Regulations for the Administration of Affairs Concerning Experimental Animals (1988) and the Hubei Regulations for the Administration of Affairs Concerning Experimental Animals (2005). Animal experiments were performed under the supervision of the Ethical Committee for Experimental Animals of Huazhong Agricultural University, Wuhan, China.

### Strain and culture conditions

The virulent Chinese *M. bovis* HB0801 strain (CCTCC # M2010040), isolated from a lung lesion in a calf with pneumonia [45], and the attenuated vaccine strain *M. bovis*-150 (CCTCC # M2011102), generated by our laboratory *in vitro* [3], were used in this study. Bacteria were cultured as previously described [2]. Stock cultures were stored as aliquots at −80°C. The *Mycoplasma bovis* PG45, *Mycoplasma agalactia* (PG2), *Mycoplasma mycoides* subsp. *capri* (PG3), *Mycoplasma ovipneumoniae* (Y98), and *Mycoplasma arginini*, and *E.coli* strains were stored in our laboratory and cultured as previously described [46]. IBRV (#AV20) and BVDV (#AV69) were purchased from the China Institute of Veterinary Drug Control and were grown according to the provided instructions.

### Extraction of whole cell proteins (WCPs)

HB0801 WCPs were extracted as previously described [47], with some minor modifications. Briefly, HB0801 cell pellets were re-suspended in PBS containing protease inhibitors (Roche, USA), and disrupted with a French press (Thermo, USA) at 20,000 lb/in^2^. After centrifugation (15,400 g, 20 min, 4°C), supernatant was treated with 15% TCA for 1 h at −20°C. Following centrifugation, the protein pellet was washed with cold acetone to remove TCA and re-suspended in lysis buffer [8 M urea, 2 M thiourea, 4% CHAPS, 2% Amidosulfobetaine-14 (ASB-14), 60 mM DTT, 40 mM Tris-HCl pH 8.8]. All the reagents were purchased from Sigma (USA). Protein concentration was measured with the 2D Quant Kit (GE healthcare, Sweden). Solubilized proteins were used immediately or stored at −80°C until use.

### Preparation of membrane-associated proteins (MAPs)

*M. bovis* MAPs were fractionated using TX-114 as previously described [48] with minor modifications. In brief, *M. bovis* HB0801 pellet was re-suspended in PBS containing 4% TX-114 (Sigma) and 1 mM PMSF (Sigma) and kept for 3-5h at 4°C after scraping. Un-lysed cells and debris were precipitated and removed by centrifugation for 15 min at 15,400 g. The supernatant was then incubated for 20 min at 37°C and then centrifuged for 5 min at 7500 g to separate the two phases. The upper aqueous phase was discarded and the lower detergent phase was reconstituted to the original volume with 1 mM PMSF in PBS for washing. After washing, the proteins in the detergent phase were re-established in 100% methanol and incubated overnight at −80°C. After centrifugation for 20 min at 15,400 g, the proteins were re-suspended in lysis buffer. Protein concentration was measured with a 2D quant kit (GE Healthcare, Sweden).

### Preparation of experimental anti-sera

Thirty-six clinically healthy 5-6 month old local calves were confirmed to be free of *M. bovis* as described previously [49]. These animals were randomly allocated among 3 groups. The 20 animals in the infected group were inoculated intratracheally with HB0801 for three consecutive days at a dose of 10^9^ cfu/calf. The 8 animals in the vaccinated group were vaccinated with the attenuated *M. bovis*-150 strain in a single intranasal dose of 10^8^ cfu/calf. The control group included 8 calves that were exposed to sterile PPLO media as a negative control. Serum samples were collected from all animals on days 0, 7, 14, 21, 28, and 35 after infection/immunization. The animals were euthanized and necropsied 40 days after infection/vaccination for gross lesion evaluation using a previously described scoring system [3]. The antibody titers of serum samples were measured by iELISA as described previously [49]. Serum samples collected on day 0 before infection or immunization were used as negative controls.

### Serum samples from the naturally infected and uninfected animals

Serum samples were collected from 46 uninfected calves and 123 calves infected with *M. bovis*, as determined by isolations conducted in our laboratory, from various cattle feedlots in China. Disease was diagnosed as described previously [49]. All serum samples were stored at −80°C for further analysis.

### Two dimensional gel electrophoresis (2-DE)

2-DE with IPG strips was conducted as previously reported [50] with some modifications. In brief, 17 cm IPG strips (pH 3-10 NL) were actively rehydrated at 50V with either *M. bovis* whole cell lysate or the TX-114 soluble fraction (380 μg/strip) in rehydration solution [7 M Urea, 2 M Thiourea, 2.5% w/v CHAPS, 2% w/v ASB-14, 40 mM Tris-HCl, pH 8.8, 65mM DTT, 0.5% IPG buffer pH 3-10 (Bio-Rad), 0.002% w/v bromophenol blue (Sigma, USA)] for 16 h at 20°C using a Protean IEF cell (Bio-Rad). Iso-electric focusing (IEF) was executed using the following program: 150 V for 3 h, 300 V for 3 h, 1000 V (gradient) for 6 h, 10000 V (gradient) for 3 h, and 10000 V for 60 kVhs (kilovolt hours). After focusing, the strips were equilibrated with 2% DTT and 4% iodoacetamide (Sigma, USA), respectively, in 10 mL equilibration solution [6 M urea,50 mM Tris-HCl pH 8.8, 30% glycerol, 2% SDS] for 15 min. The second separation was performed on 10% gels (SDS-PAGE) at 12°C for 3 h at 50 V followed by 12 h at 100 V using the Protean II xi multi-cell with 2-D conversion kit (Bio-Rad, USA). All experiments were run in triplicate. Proteins were stained on the first gel with 0.15% Coomassie brilliant blue R-250 (CBB R-250- Bio-Rad, USA), scanned with a GS-800 Calibrated Densitometer (Bio-Rad), and evaluated with the PD Quest Basic 8.0 program (Bio-Rad, USA). Proteins from other gels were subjected to an immunoblotting assay.

### Immunoblotting assay

Western blot analysis of the proteins separated by 2-DE was performed as described previously [47] with some modifications. Briefly, proteins separated on 2-DE gels were electro-blotted onto PVDF membranes, blocked with 5% dried skimmed milk powder, and probed for 1 h at room temperature with pooled sera (1:500) collected from all experimentally infected calves 35 days after infection. Pooled sera collected on day 0 before infection were used as negative controls. Immunoblots were established with horseradish peroxidase (HRP)-conjugated goat anti-bovine IgG (Pierce, USA, 1:5000) for 1 h, developed with super signal west femto chemiluminescent substrate (Pierce, USA), and visualized on the Chemiluminescence & Fluorescence DNr Bio-imaging system (DNr, Israel).

### Matrix-assisted laser desorption/ionization time of flight (MALDI-TOF) mass spectrometry (MS)

Antigenic proteins were removed from the gel and digested with trypsin. In brief, gels were first treated with 200-400 μL of destaining solution (100 mM NH_4_HCO_3_ in 30% ACN) and lyophilized. Peptide extraction was performed three times with 60% ACN/0.1% TFA, and proteins were freeze dried after gel digestion in 5 μL (2.5-10 ng/μL) of trypsin at 37°C overnight. Dry samples were reconstituted in 20 % ACN (2 μL) and loaded on a 384-well Opti-TOF (123 mm × 81 mm) stainless steel plate. The samples in the plate were then overlaid with 0.5 μL CHCA in 50% ACN and 0.1% TFA. Mass spectrometry and MS/MS data for the recognition of proteins were generated with a MALDI-TOF appliance (4800 plus proteomics analyzer, Applied Biosystems). The conditions were fixed using the 4000 Series Explorer software (Applied Biosystems). The MS ranges were documented in reflector mode at masses ranging from 800 to 4000 Da with a focal mass of 2000 Da. A CalMix5 standard was used to adjust the MS equipment (ABI 4700 Calibration Mixture). For a single core MS spectrum, 25 subspectra with 125 shots per subspectrum were collected using a random search pattern. For MS standardization, trypsin autolysis peaks ([M+H]+842.5100 and 2,211.1046) were used as internal controls, and up to 10 of the strongest ion signals were chosen as precursors for MS/MS analysis, excluding the autolysis peaks of trypsin and matrix ion signals. In MS/MS positive ion mode, for a single core MS spectrum, 50 subspectra with 50 shots per subspectrum were taken with a random search pattern. Collision energy was 2 kV, collision gas was air, and default calibration was set with Glu1-Fibrino-peptide B ([M+H]+ 1,570.6696) spotted onto the Cal 7 positions of the MALDI target. Collective peptide mass fingerprinting (PMF) and MS/MS inquiries were accomplished by the MASCOT search engine 2.2 (Matrix Science, Ltd., U.S.) in the GPS-Explorer Software 3.6 (Applied Biosystems) from the NCBI database (Taxonomy: NCBI_Bacteria 5910423, 12/3/2010, NCBI_Mycoplasma 198866, 22/5/2014). The search criteria used in this study were trypsin specificity, one missed cleavage, carbamidomethylation of cysteines as fixed modifications, and oxidation of methionine residues as dynamic modifications. The peptide mass tolerance was set to ±100 ppm and the MS/MS fragment tolerance was set to ± 0.4 Da. A GPS Explorer protein confidence interval ≥ 95% (protein score C.I. %) was used for further validation. The identified protein sequences were obtained from NCBI. Upon comparison to a cluster of orthologous groups (COGs) database using RPS-BLAST, functional classification of proteins was determined, and subcellular localization of identified proteins was predicted with the PSORTb database.

### Prediction of T- and B-cell epitopes in *M. bovis* antigenic proteins

The amino acid sequences of antigenic *M. bovis* HB0801 proteins were subjected to T- and B- cell epitope prediction with tools cited in the Immune Epitope Database and Analysis Resource (IEDB-AR), a database of experimentally identified B- and T-cell epitopes (http://tools.immuneepitope.org/mhci/), as described previously [28]. Modeling of 3D structure templates was performed with the SWISS-MODEL Workspace (http://swissmodel.expasy.org) and ElliPro (http://tools.immuneepitope.org/tools) [51, 52]. Templates were acquired for *M. bovis* proteins by modeling their sequences in the FASTA format. To predict B-cell conformational epitopes, a modeled template of each protein was submitted to Ellipro using conditions similar to those reported previously for cattle pathogens [28].

### Site-directed mutagenesis, cloning, expression, and purification of HB0801 antigens

Eight highly conserved antigenic proteins (Table 3), with large numbers of predicted T- and B- cells epitopes, were selected for subsequent analysis*. The complete genes of these M. bovis proteins were* amplified by overlap extension PCR for the site-directed mutagenesis using the primers in Table S1 and *pfu* DNA polymerase (Thermo, USA) to avoid the translational hurdle of differences in UGA codon usage between mycoplasmas and *E. coli*. The complete genes, with single nucleotide changes in the UGA codon (UGA→UGG), were cloned into pET-30a (+) vectors. The constructs were confirmed by nucleotide sequencing (Sangon Company, China). Recombinant plasmids were transformed into *E. coli* BL21 (DE3) (Novagen, USA) competent cells for expression. A single clone of each recombinant plasmid was inoculated in 5 mL of Luria-Bertani (LB) medium containing 120 μg/mL kanamycin and incubated in a shaker (37°C, 250 rpm) until the OD_600_ reached 0.6. Recombinant protein expression was induced with a 0.4 mM final concentration of IPTG, and the culture was allowed to grow at 30°C for 4 h. After extraction and washing with PBS, the recombinant proteins were purified by nickel affinity chromatography (GE Healthcare, Sweden). Protein concentrations were measured by the BCA method (Thermo, USA) and verified with 12% SDS-PAGE.

### Characterization of promising *M. bovis* antigens

The 8 purified recombinant proteins (2 μg) were separated by 12% SDS-PAGE and blotted onto a PVDF membrane. After blocking, recombinant proteins were probed with serum samples collected from the following groups of animals: (a) *M. bovis* HB0801-infected calves 35 days after infection; (b) *M. bovis*-150-immunized calves 35 days post-vaccination; and (c) naturally infected calves.

In addition, the eight recombinant antigenic proteins (Table 3) and *M. bovis* WCPs were characterized with iELISAs. iELISA conditions were optimized for *M. bovis* WCPs and each recombinant protein as described elsewhere [49]. In brief, 96-well microtitre plates were coated overnight at 4°C with 125 ng of WCPs and 250 ng of each purified recombinant protein diluted in 100 μL sodium carbonate buffer (pH 9.6) and washed with PBS containing 0.05% Tween 20 (PBST). After blocking, the plates were probed for 1 h at 37°C with sera at various dilutions collected from the experimental and naturally infected groups of calves described above. After washing with PBST, plates were incubated for 1 h at 37°C with goat anti-bovine IgG-HRP (1:5000) (Southern Biotech Co. USA) and washed with PBST followed by the addition of tetramethylbenzidine (TMB)/H_2_O_2_ (Wuhan Keqian Biological Co., Ltd, China) as a substrate. The reaction was stopped after 10 min and OD values were obtained with a microtiter plate reader (BioTek, USA) at 630 nm (OD_630_). Cut-off OD_630_ values were determined for WCP and recombinant protein-based iELISAs using serum samples from the 46 uninfected animals. Mean OD_630_ values and standard deviations were calculated, and cut-off values were set at mean+2SD.

### Production of mouse polyclonal antibodies against MAPs

Ten female BALB/c mice (5 weeks old) were purchased from the China Hubei Provincial Center for Disease Control and Prevention to produce polyclonal antibodies against rMbovP579 and rMbovP739. Mice were divided into three groups (4 mice in each protein group and 2 mice in the negative control group). Mice in each protein group were immunized subcutaneously with 100 μg in 200 μL of purified recombinant protein mixed with an equal volume of Freund's complete adjuvant (Sigma, USA). Two subsequent boosters, each with the same amounts of protein and Freund's incomplete adjuvant (Sigma, USA), were administered at intervals of 2 weeks. The serum titer of the polyclonal antibodies was evaluated after each immunization. When titers increased significantly, the mice were euthanized; antiserum to each protein was collected and stored at −20°C for further use. Sera from each inoculated mouse on day 0 and serum samples from uninfected mice were used as negative controls.

### Specificity of the two lipoproteins

In the Western blot analysis, the specificities of *M. bovis* MbovP579 and MbovP739 in WCPs from the following organisms, including related mycoplasma species, were determined using the mouse anti-rMbovP579 and anti-rMovP739 polyclonal antibodies: 1) *M. bovis* HB0801; 2) attenuated vaccine strain *M. bovis*-150; 3) *M. bovis* PG45 (ATCC 25523); 4) *Mycoplasma agalactiae* PG2; 5) *Mycoplasma mycoides* subsp. *capri* (PG3); 6) *Mycoplasma ovipneumoniae* (Y98); 7) *Mycoplasma arginini*; and 8) *E.coli* strain BL21-DE_3_ as a negative control. The protein concentration of each sample was measured using the BCA method (Thermo, USA). The specificities of MbovP579 and MbovP739 were also determined by iELISA using reference antiserum against common cattle pathogens, including IBRV and BVDV. Each protein sample (25 μg) was resolved on a 12% SDS-PAGE gel and blotted onto a PVDF membrane at 15V for 45 min using a trans-blot semi-dry transfer cell (Bio-Rad). Homology of the MbovP579 amino acid sequence with other mycoplasma sequences was analyzed using the NCBI database (Table 4). All pathogens described above were identified using specific PCR before these experiments.

### Prediction of MbovP579 secretion

*In silico* analysis was used to predict whether MbovP579 was a secretory or non-secretory protein. Both classical and non-classical secretion were included. Prediction of classical secretion and identification of the presence of signal peptides were conducted using SignalP 4.1 (http://www.cbs.dtu.dk/services/SignalP) and PRED-LIPO (http://bioinformatics.biol.uoa.gr/PRED-LIPO/) as reported previously [41, 42]. SecretomeP 2.0 (http://www.cbs.dtu.dk/services/SecretomeP) was used to predict nonclassical secretion [43]. The predictions were executed at a default cutoff value of 0.5. Predicted scores of 0.5 and above were considered indicative of secretion.

### Establishment of rMbovP579-based iELISA

An iELISA was established as described previously [49]. Briefly, 96-well ELISA plates were coated with rMbovP579 serially diluted two-fold from a concentration of 2 μg to 0.97 ng/well and incubated at 4°C overnight. Sera were added at two-fold serial dilutions of 1:25, 1:50, 1:100, 1:200, 1:400, 1:800, and 1:1600 (v/v). Commercial goat anti-bovine IgG-HRP secondary antibody was diluted to 1:3000, 1:5000, and 1:8000 (v/v). TMB/H_2_O_2_ was added as substrate to the wells and incubated for 10 min, and the OD_630_ values were measured with a microplate reader. To compare the performance of iELISA and standard tests, serum samples from the 20 experimentally infected calves, the 8 uninfected experimental calves, and the 123 naturally infected and 46 uninfected animals from various feedlots were analyzed using this iELISA; sensitivity and specificity were determined based on rMbovP579 and Kappa agreement. The cut-off value was defined as the value where both sensitivity and specificity were highest in receiver operating characteristic (ROC) analysis. Additionally, antisera against IBRV and BVDV purchased from the China Institute of Veterinary Drug Control were evaluated with the iELISA to confirm its specificity.

### Comparison of the rMbovP579-based iELISA and a commercial Kit

Serum samples from 123 naturally infected animals and 20 experimentally infected animals were separately subjected to the rMbovP579-based iELISA and the commercial kit (BioX, Belgium) under standardized conditions, and the sensitivity of the two assays, as well as the degree of agreement, were determined. Additionally, the analytic sensitivities of the rMbovP579-based iELISA and commercial kit were individually evaluated with 93 positive serum samples excluding samples with OD_630_ values ≥2.2±0.2 or ≤0.8±0.2. The sera were two-fold serially diluted from 1:100 to 1:102,400 and tested in parallel using both methods. The highest dilution for which the assay returned a positive result indicated the analytic sensitivity, while the proportion of true positives (determined by *M.bovis* isolation, the gold standard method) detected indicated the diagnostic sensitivity.

### Data availability

The MS/MS proteomics data have been deposited in the ProteomeXchange Consortium (http://proteomecentral.proteomexchange.org) via the PRIDE [53] partner repository with the dataset identifier PXD003479.

### Statistical analysis

The Kappa agreement coefficient between the assays, cut-off values, and sensitivity and specificity based on ROC analysis were determined online using EpiTools. The differences in analytic sensitivity between the rMovP579-based iELISA and commercial kit were analyzed using the Chi-square or Fisher's exact test. p values below 0.05 were considered statistically significant; *p<0.05, **p<0.01.

## SUPPLEMENTARY MATERIAL TABLES AND FIGURE


