# Supplementary material for: Immunoproteomic identification of MbovP579, a promising diagnostic biomarker for serological detection of Mycoplasma bovis infection
Source: Oncotarget. 2016 Jun 2;7(26):39376–95. doi: 10.18632/oncotarget.9799 (PMC5129939; doi:10.18632/oncotarget.9799)
Supplement: Supplementary file 1 [file oncotarget-07-39376-s001.pdf]

Immunoproteomic identification of MbovP579, a promising diagnostic biomarker for serological detection of *Mycoplasma bovis* infection

Supplementary Material

Table S1. List of primers used in overlap extension PCR for site-directed mutagenesis

Primer Names	Sequences (5′to 3′)	Description
116-A1	5′-CGGGGTACCATGAATAAGAAAACTAAATTAGTTATGTTATCAATGCCTA	Forward primer with a *Kpn*1 restriction site underlined for cloning into pET-30a(+)
116-A2	5′-CCGGAATTCCTACCAGGTGTATTTATATTCATATTCTTTATCAGAAGTT′	Reverse primer with a *Eco*R1 restriction site for cloning into pET-30a(+) and for site UGA$^{130395-7}$ -UGG directed mutagenesis
275-a1	5′-ATGAAAAAAATCAAAATATTAAGTAGTATTGGAATATTAG	Forward primer
275-a2	5′-CATTTGTGTTTTTTATCCAATTATTGATTTCTTTG	Reverse primer for site UGA$^{315721-3}$-UGG directed mutagenesis
275-b1	5′-TAGCAAAGAAATCAATAATTGGATAAAAAAACACAAATGATGC	Forward primer for site UGA$^{315721-3}$-UGG directed mutagenesis
275-b2	5′-TTTAAACCAATATTTTTCCATGTTATGGGCGCATAAT	Reverse primer for site UGA$^{316111-3}$-UGG directed mutagenesis
275-c1	5′-ATTATGCGCCCATAACATGGAAAAATATTGGTTTAAAT	Forward primer for site UGA$^{316111-3}$-UGG directed mutagenesis
275-c2	5′-TTAATTGTTTTTTTGATTGTGAACAATGAAACTGATTTTTGAA	Reverse primer
275-M1	5′-CGGGGTACCATGAAAAAAATCAAAATATTAAGTAGTATTGGAATATTAG	Forward primer with a *Kpn*1 restriction site for cloning into pET-30a(+)
275-M2	5′-CCGGAATTCTTAATTGTTTTTTTGATTGTGAACAATGAAACT	Reverse primer with a *Eco*R1 restriction site for cloning into pET-30a(+)
579-a1	5′-ATGAGTAAGAAAAATAAATTAATGATTGGGCTTTCATCTACTGCT	Forward primer
579-a2	5′-GAAGCCATAGTATTCCATTGTGGCTGACCA	Reverse primer for site UGA$^{683963-5}$-UGG directed mutagenesis
579-b1	5′-GGTCAGCCACAATGGAATACTATGGCTT	Forward primer for site UGA$^{683963-5}$-UGG directed mutagenesis
579-b2	5′-GTGAACTTTCAAATTCACCCCATAATTTTTGTACTTCACT	Reverse primer for site UGA$^{684488-90}$-UGG directed mutagenesis
579-c1	5′-GTGAAGTACAAAAATTATGGGGTGAATTTGAAAGTTCACA	Forward primer for site UGA$^{684488-90}$-UGG directed mutagenesis
579-c2	5′-TTCTCTTAAGAATAATAGCCATTGTTTATTATCACTAGCTTC	Reverse primer for site UGA$^{685253-5}$-UGG directed mutagenesis
579-d1	5′-AGCTAGTGATAATAAACAATGGCTATTATTCTTAAGAGAAGAT	Forward primer for site UGA$^{685253-5}$-UGG directed mutagenesis
579-d2	5′-TTCATTAGATTTATTCCATTTACCAGGCACAGC	Reverse primer for site UGA$^{685472-4}$-UGG directed mutagenesis
579-e1	5′-GCTGTGCCTGGTAAATGGAATAAATCTAATGA	Forward primer for site UGA$^{685472-4}$-UGG directed mutagenesis

579-e2	$5'$-TTATTTAAGAATTTGACTGCTTGATGCAATAATTGATCCAATA	Reverse primer
579-M1	$5'$-CGGGGTACCATGAGTAAGAAAAATAAATTAATGATTGG	Forward primer with a *Kpn*1 restriction site for cloning into pET-30a(+)
579-M2	$5'$-CCGGAATTCTTATTTAAGAATTTGACTGCTTGATGC	Reverse primer with a *Eco*R1 restriction site for cloning into pET-30a(+)
0739A1	$5'$-CGGAATTCTTGAAAAAAATTTTTATTAAAGCTGGTGCAATTTTATTAC	Forward primer with a *Eco*R1 restriction site for cloning into pET-30a(+)
0739A2	$5'$-CATTCTTAGCTTTTAAAGCATACCAAATTGATTGTTCAGG	Reverse primer for site UGA$^{872192-4}$-UGG directed mutagenesis
0739B1	$5'$-CTGAACAATCAATTTGGTATGCTTTAAAAGCTAAG	Forward primer for site UGA$^{872192-4}$-UGG directed mutagenesis
0739B2	$5'$-TGTAGTCTTTCCATAATTCTTGGAATTTTTTCTTA	Reverse primer for site UGA$^{872510-2}$-UGG directed mutagenesis
0739C1	$5'$-TAAGAAAAAATTCCAAGAATTATGGAAAGACTACA	Forward primer for site UGA$^{872510-2}$-UGG directed mutagenesis
0739C2	$5'$-CTAAAGATTTGCCATGAACCCCATTCTTTTG	Reverse primer for sites UGA$^{872624-6}$and UGA$^{872633-5}$-UGG directed mutagenesis
0739D1	$5'$-TCATGGCAAATCTTTAGATTCCAAAGTGCTATA	Forward primer for site UGA$^{872633-5}$-UGG directed mutagenesis
0739D2	$5'$-CAGAAACCTTGTTTTTGCCTGTGTATATCCATTTAA	Reverse primer for site UGA$^{872921-3}$-UGG directed mutagenesis
0739E1	$5'$-TTTCTGGCGAAGAAGAAGATAACTGGTACACA	Forward primer for site UGA$^{872969-71}$-UGG directed mutagenesis
0739E2	$5'$-GCAAGCTTTTATTGTTTTTTAGTTTGTTCAAATGATTTTATTAGTTCT	Reverse primer with a *Hin*d III restriction site for cloning into pET-30a(+)
0106A1	$5'$-CGGTACCATGACAGCAGAATCAAAATG	Forward primer with a *Kpn*1 restriction site for cloning into pET-30a(+)
0106A2	$5'$-TATTTAAGCCCTTCATCTTTCCAAG	Reverse primer for site UGA$^{115114-6}$-UGG directed mutagenesis
0106B1	$5'$-GGGCTTAAATACGAAGGCGAAGTTGCTGA	Forward primer
0106B2	$5'$-TATTTAAACAAACACCACCCCAAAATTCTTTTTCAACAAT	Reverse primer for site UGA$^{115258-60}$-UGG directed mutagenesis
0106C1	$5'$-GGTGTTTGTTTAAATATTGGATGTATTCCTACCAAA	Forward primer
0106C2	$5'$-GCTTTACGTTCATGCATTTTAACCCATGATT	Reverse primer for site UGA$^{115402-3}$-UGG directed mutagenesis
0106D1	$5'$-CGTAAAGCCAAAGTTGTTGCAAAGCT	Forward primer
0106D2	$5'$-ATAAAGCACTTCTAGCTGCTTCCCAAATAATTTCATTAA	Reverse primer for site UGA$^{116512-4}$-UGG directed mutagenesis
0106E1	$5'$-AATTATTTGGGAAGCAGCTAGAAGTGCTTTA	Forward primer for site UGA$^{116512-4}$-UGG directed mutagenesis
0106E2	$5'$-CGGATCCTTAGTTGTTTTTTCTTTCGTTTAATTTTTC	Reverse primer with a *Bam*H1 restriction site for cloning into pET-30a(+)
126-a1	$5'$-ATGAACAGACAATTGTTAGACAGAATATTTGCTGAAAAAA	Forward primer
126-a2	$5'$-AGTGGTTTGAACATTTGAATACCATAGTCTAGTCTGTGGA	Reverse primer for siteUGA$^{139224-6}$-UGG directed mutagenesis
126-b1	$5'$-CACAGACTAGACTATGGTATTCAAATGTTCAAACCACTGA	Forward primer for siteUGA$^{139224-6}$-UGG directed mutagenesis
126-b2	$5'$-TCAGTCATGCCAGGAATAATCCAGCTTATTAATTTTTCAT	Reverse primer for siteUGA$^{139584-6}$-UGG directed mutagenesis
126-c1	$5'$-ATGAAAAATTAATAAGCTGGATTATTCCTGGCATGACT	Forward primer for siteUGA$^{139584-6}$-UGG directed mutagenesis
126-c2	$5'$-TTACATTGCTTCTTCTGGACGTGTCAGAACA	Reverse primer

126-M1	5′-CGGGGTACCATGAACAGACAATTGTTAGACAGAATATTT	Forward primer with a *Kpn*1 restriction site for cloning into pET-30a(+)
126-M2	5′-CGGAATTCTTACATTGCTTCTTCTGGACGTGTCA	Reverse primer with a *Eco*R1 restriction site for cloning into pET-30a(+)
212-a1	5′-ATGAATATTAATCAAAAAGTTGTTGCATCTATG	Forward primer
212-a2	5′-CTATCTCTGCTAATCCATTTTGGATGATC	Reverse primer for site UGA$^{246100-2}$-UGG directed mutagenesis
212-b1	5′-CTGATCATCCAAAATGGATTAGCAGAGATAGAC	Forward primer for site UGA$^{246100-2}$-UGG directed mutagenesis
212-b2	5′-GATTATCTAACTCAACTAACCATTCTTCATAGCTTTTTGC	Reverse primer for site UGA$^{246853-5}$-UGG directed mutagenesis
212-c1	5′-ATGGTTAGTTGAGTTAGATAATCATAAAAAACAATTTCCATGGATTCATTCATTATTA	Forward primer for sites UGA$^{246853-5}$ and UGA$^{246892-4}$-UGG directed mutagenesis
212-c2	5′-TTGTTATATTTACTTAATTTATACCATAGTGGATCGTTTGATGCT	Reverse primer for site UGA$^{247735-7}$-UGG directed mutagenesis
212-d1	5′-GCATCAAACGATCCACTATGGTATAAATTAAGTAAATATA	Forward primer for site UGA$^{247735-7}$-UGG directed mutagenesis
212-d2	5′-TTAAGCATTTTTTGACAAGAATTGTTTTACTTTATCAACT	Reverse primer
212-M1	5′-CGGGGTACCATGAATATTAATCAAAAAGTTGTTGCATCTAT	Forward primer with a *Kpn*1 restriction site for cloning into pET-30a(+)
212-M2	5′-CCGGAATTCTTAAGCATTTTTTGACAAGAATTGTTTTACTTTATCAACT	Reverse primer with a *Eco*R1 restriction site for cloning into pET-30a(+)
789-a1	5′-ATGAAATTATTTGAATCAATATCATCAAAAAGAGATAATA	Forward primer
789-a2	5′-TCTTTATGTCTTCCCAAGCTTTATCACTA	Reverse primer for site UGA$^{922440-2}$-UGG-directed mutagenesis
789-b1	5′-TAGTGATAAAGCTTGGGAAGACATAAAGAA	Forward primer for site UGA$^{922440-2}$-UGG-directed mutagenesis
789-b2	5′-AAGCGGCATTCTCCAAACTAATTCG	Reverse primer for site UGA$^{922485-7}$-UGG-directed mutagenesis
789-c1	5′-GAATTAGTTTGGAGAATGCCGCTTG	Forward primer for site UGA$^{922485-7}$-UGG-directed mutagenesis
789-c2	5′-TTAAAGTAAACTTAATTCAGTTAAAGTTTTAACCATAATTCC	Reverse primer
789-M1	5′-CGGGGTACCATGAAATTATTTGAATCAATATCATCAAAAA	Forward primer with a *Kpn*1 restriction site for cloning into pET-30a(+)
789-M2	5′-CCGGAATTCTTAAAGTAAACTTAATTCAGTTAAAGTTTTAACCAT	Reverse primer with a *Eco*R1 restriction site for cloning into pET-30a(+)

Table S2. Conservation of the MbovP579 in all the sequenced Chinese strains of *M. bovis*

Names	Isolation time<br>Year-month-date	Tissues	Location in China	Identity (%)
M.bovis HBJS080514	2008.05.14	lung	Hubei Jingshan	100
M.bovis HBSZ080607	2008.06. 07	lung	Hubei Suizhou	100
M.bovis HBEZ080610	2008.06.10	lung	Hubei Ezhou	100
M.bovis AHBZ080610	2008.06.10	Lung	Anhui Bozhou	100
M.bovis HBEZ08071003	2008.07.10	lung	Hubei Ezhou	99.85
M.bovis HBEZ08071008	2008.07.10	lung	Hubei Ezhou	99.85
M.bovis HBEZ08071002	2008.07.10	lung	Hubei Ezhou	100
M.bovis HBXZ08071102	2008.07.11	lung	Hubei Xinzhou	99.86
M.bovis HBXZ08071101	2008.07.11	Lung	Hubei Xinzhou	99.86
M.bovis HBFX081205	2008.12.05	Lung	Hubei Fangxian	99.86
M.bovis HNYL090225	2009.02. 25	lung	Henan Yanling	99.86
M.bovis FJXM091017	2009.10.17	lung	Fujian Xianmen	99.85
M.bovis HNYL091112	2009.11.12	lung	Henan Yanling	100
M.bovis HNLJ091225	2009.12.25	lung	Hunan Lianjiang	100
M.bovis HNYJ100203	2010.02.03	lung	Henan Yanjing	100
M.bovis HBHA100501	2010.05.01	Throat	Hubei Hongan	100
M.bovis HBZX100630	2010.06. 30	lung	Hubei Zhongxiang	100
M.bovis NMKEQ100717	2010.07.17	Lung	Neimeng Keerqin	99.86
M.bovis NMYL100719	2010. 07.19	lung	Neimeng Yuliang	100
M.bovis HBDY100723	2010.07.23	lung	Hubei Daye	100
M.bovis JXJX101006	2010.10.06.	lung	Jiangxixinyu	100
M.bovis HNKF101010	2010.10.10	lung	Henan Kaifeng	99.85
M.bovis HNKRQ110428	2011.04.28	Lung	Henan Keerqin	100
M.bovis HNZMD110528	2011.05.28	lung	Henan Zhumadian	100

M.bovis JXGA110719	2011.07.19.	lung	Jiangxi Gaoan	100
M.bovis GZSZ120527	2012.05.27	lung	Guangzhou Shenzhen	100
M.bovis HBTS120615	2012.06.15	lung	Hubei Tongshan	100
M.bovis HBJX120715	2012.07.15	Lung	Hubei Jiangxia	100
M.bovis HBSY130124	2013.01.24.	lung	Hubei Shayang	100
M.bovis HBWX130312	2013.03.12	Joint	Hubei Wuxue	100
M.bovis HBYC130315	2013.03.15	Milk	Hubei Yichang	99.85
M.bovis GZSZ130410	2013.04.01	Lung	Guangzhou Shenzhen	100
M.bovis HBHS130614	2013.06.14	lung	Hubei Huangshi	100
M.bovis SD130624	2013.06.24	Lung	Shandong	100

Table S3. Cut-point results for target sensitivity of rMbovP579-based iELISA

Target Se	Cut-off point	Se	Se Lower 95% CI	Se Upper 95% CI	Sp	Sp Lower 95% CI	Sp Upper 95% CI
0.999	0.173	1	0.97	1	0.174	0.091	0.307
0.995	0.173	1	0.97	1	0.174	0.091	0.307
0.99	0.183	0.992	0.955	0.999	0.239	0.139	0.379
0.98	0.192	0.984	0.943	0.996	0.261	0.156	0.403
0.95	0.323	0.951	0.898	0.977	0.696	0.552	0.809
**0.9**	**0.442**	**0.902**	**0.837**	**0.943**	**0.978**	**0.887**	**0.996**
0.8	0.717	0.805	0.726	0.865	1	0.923	1

Se, Sensitivity; Sp, Specificity; 95% CI, 95% Confidence Interval.

Table S4. Performance of rMbovP579-based iELISA in the detection of *M. bovis* natural infection

		Diagnosed by *M.bovis* detection (Gold standard test)		
		Positive	Negative	Total
rMbovP579-based iELISA	Positive	111	1	112
	Negative	12	45	57
	Total	123	46	169

Table S5. Performance of rMbovP579-based iELISA in the detection of *M. bovis* experimental infection

		Diagnosed by *M.bovis* detection (Gold standard test)		
		Positive	Negative	Total
rMbovP579-based iELISA	Positive	20	0	20
	Negative	0	8	8
	Total	20	8	28

Table S6. Comparison of rMbovP579-based iELISA and the commercial kit to detect *M. Bovis* natural infection

		Commercial iELISA kit		
		Positive	Negative	Total
rMbovP579-based iELISA	Positive	39	72	111
	Negative	0	12	12
	Total	39	84	123

Table S7: MHC class I T cell epitopes (nine-mer)

Protein	Intermediate affinity binding		High affinity binding	
	**Sequence**	**Position**	**Sequence**	**Position**
Lipoprotein (MbovP579)	VIYEEPAGF	674-682	KESLATKSL	342-350
	IYNKFKESL	337-345	RANSNTENL	156-164
	STFENLEEL	249-257	VIYSQGPSL	566-574
	KFLTSLEKI	592-600	NYGSLASRL	111-119
	YIVHTYNEL	656-664	EELLNFANI	255-263
	IYSQGPSLI	567-575	FEKTDTSKI	644-652
	DESEVQKLW	222-230		
	KELSSKGTL	536-544		
	FMIPMLKSI	170-178		
	TYNELKEAV	660-668		
	KEAVTNKNV	665-673		
	ISSSTFENL	246-254		

	GENTVITEL	90-98		
	FDKEIIGSI	711-719		
	SIIASSSQI	718-726		
	FESSQQDAV	233-241		
	QWNTMASLI	54-62		
	TKHEYAFGI	364-372		
	TAGYRHNFI	375-383		
	QDAVKKLTI	238-246		
	LEDFDKEII	708-716		
	SYQTKHEYA	361-369		
	NNGTFMIPM	166-174		
	SYKSLDSAT	453-461		
	KNDKHFLPV	70-78		
	YAMINADAN	295-303		
Putative Lipoprotein (MbovP739)	TELDRNKEF	591-599	GEYQLLKKV	95-103

	TKKTFSKSI	144-152	LNMLRSAQL	544-552
	GAYQNKITM	393-401	NEYGEYQLL	92-100
	FELDKDKNV	310-318	IENENVEGI	202-210
	QIFRFQSAI	378-386	SAISFAASV	384-392
	QQNTFFKEL	290-298		
	EEEDNWYTI	484-492		
	LPAAAGLSV	13-21		
	NHPYFQQSI	405-413		
	NQNKQLLDI	130-138		
	IFHEGGSSI	445-453		
	DKNVKYNLI	315-323		
	GYIMPLASV	497-505		
	NDNTISGEV	276-284		
Putative Lipoprotein (MbovP116)	RPNDNYGYL	91-99	NEGIKKGFV	196-204
	KEYEYKYTW	320-328	VTPKKFIYL	114-122

	SSYEKVLEV	65-73	VNLSFTYTV	204-212
	VMLSMPIAL	8-16		
	SYISLKNAG	270-278		
	MPIALFAPV	12-20		
	TEIINDKLL	160-168		
	LYFSKTRNL	140-148		
	IEKSSATDV	251-259		
Putative Lipoprotein (MbovP275)	TYVDIKNAL	61-69	YENNRKSVI	39-47
	NHYLDDSFI	244-252	SYKTFKNSI	94-102
	YHFYLTKYV	329-337	KYSFLKTYV	55-63
	EESLKDISI	169-177	ISIDFENSV	175-183
	SAFDNLLVL	115-123		
	KYENNRKSV	38-46		
	SYKTFKNSI	94-102		
	TENDKNHYL	239-247		

	SSIGILAPV	8-16		
	FPKTIINQL	135-143		
	SFLKTYVDI	57-65		
	LAPVLAIPL	13-21		
	SYSLIGSHA	315-323		
	KKIKILSSI	2-10		
Dihydrolipoamide dehydrogenase (DLD)	YEGEVADEF	57-65	VSKYKFATL	440-448
	IYGATSTKL	300-308	TEEGVAKFV	175-183
	SHANRMKFL	206-214	MYASMGTKV	258-266
	REVKVDKFL	354-362	AYIHAVTAV	385-393
	AMLRSTHAL	112-120	SEGLAEVGI	340-348
	KFATLGKAI	444-452	EEKITAEVI	322-330
	KLMTSREAI	225-233	HPHPTFNEI	506-514
	TIIQREDRL	267-275	VEKEFWGGV	93-101

	IELGARREV	348-356		
	VIGVEFAQM	250-258		
	VAYIHAVTA	384-392		
	NMLAHVAYI	379-387		
	REIEVNGKV	186-194		
	REDRLLPGI	271-279		
	KILKTESKI	289-297		
	KEQGLDFFV	432-440		
	KEEKITAEV	321-329		
	ATGSHANRM	203-211		
XAA-Pro aminopeptidase (pepP)	IAFIKGQEL	111-119	YEKLISWII	142-150
	EEIQAMQEV	126-134	IVYDEFDRL	94-102
	PEQEKILEI	234-242	AYEKLISWI	141-149
	ISLKAYEKL	137-145		
	QTRLWYSNV	24-32		

	TEKQIATKL	154-162		
	QIATKLNHL	157-165		
	AEKNIDCIV	11-19		
	FAEKNIDCI	10-18		
	IDKICRDYI	263-271		
Transketolase (Tkt)	LAYFKALNF	201-209	TNYDFSNIL	331-339
	MSFYSMMHF	69-77	SMMHFLGLL	73-81
	YELVMGLNY	483-491	TAINNGINL	401-409
	SNVDLLAYF	196-204	RSIPNLLVI	469-477
	SFYSMMHFL	70-78	HPETDAFDF	99-107
	KYNKIDGHF	604-612	TYIMVHNII	236-244
	ASVMKLNKF	169-177	YSMMHFLGL	72-80
	MQAIALDSI	11-19	ASMSFYSMM	67-75
	SYDNIDKAI	218-226	SEFMAASLV	518-526
	FKALNFNVI	204-212	AYNNTSTQV	492-500

ISPIMYAVV	33-41	HEALQIASV	163-171
EQVTVLRSI	463-471	VELKNHGII	551-559
IHSLLLETI	319-327	DYFKAFSEI	349-357
IGLENKAPF	273-281		
NHGIIANVI	555-563		
SMGFNVNSI	628-636		
VELAYKTAV	543-551		
TKVRFADSI	376-384		
AEKKMSLKI	128-136		
SVMKLNKFI	170-178		
IVDFSYPFL	357-365		
SILDYGQRI	383-391		
DELAIKLKL	575-583		
KVIDNYTYV	142-150		
MYAVVAKHM	37-45		

	KVVASMQAI	6-14		
	MKLNKFILI	172-180		
	TYIMVHNII	236-244		
	PETDAFDFV	100-108		
	VYEYGRTIM	287-295		
	YKLSKYNKI	600-608		
	LGLLSLDEM	76-86		
	YEYGRTIMA	288-296		
	FSYPFLIGG	360-368		
Leucyl aminopeptidase (pepA)	VMIGKGITF	227-235	EEIELVPFI	118-126
	SAAMFLEEF	413-421	NELVWRMPL	376-384
	VYFSKKESL	48-56	IEELKMGLL	190-198
	EELKMGLLL	191-199	DETKCNFLI	23-31
	IMVKTLTEL	445-453		
	KAAENANEL	370-378		

SEVPQGIMV	439-447
KFDMSGSAI	252-260
TNFARDLQI	147-155
EKVEIVKVI	86-94
QYKNLKVTV	176-184
DEKEEIELV	115-123
LDKAFAKNI	384-392
TEYHEKNEA	38-46
LPDSVWVAM	294-302
SMVSMKFDM	247-255

The predicted epitope result is based on the scores of antigenic peptide processing (proteasomal cleavage and TAP transport) and MHC binding affinity. Score is categorized as intermediate binding affinity (IC50 < 500 nM) and high binding affinity (IC50 < 50 nM).

Table S8: MHC class II T cell epitopes (fifteen-mers)

Protein	Intermediate affinity binding		High affinity binding	
	Sequence	Position	Sequence	Position
Lipoprotein (MbovP579)	LSSTAIPLLAAVSAK	11-25	GLSSTAIPLLAAVSA	10-24
	MIGLSSTAIPLLAAV	8-22	IPMLKSIQVMSANAP	172-186
	LKSIQVMSANAPVLQ	175-189	IGLSSTAIPLLAAVS	9-23
	MIPMLKSIQVMSANA	171-185	PMLKSIQVMSANAPV	173-187
	FMIPMLKSIQVMSAN	170-184		
	LMIGLSSTAIPLLAA	7-21		
	MLKSIQVMSANAPVL	174-188		
	TFMIPMLKSIQVMSA	169-183		
	YGSLASRLASSEMRD	112-126		
	NYGSLASRLASSEMR	111-125		
	FNYGSLASRLASSEM	110-124		
	AFNYGSLASRLASSE	109-123		

	KLMIGLSSTAIPLLA	6-20	
	LAFNYGSLASRLASS	108-122	
	STAIPLLAAVSAKCG	13-27	
Putative Lipoprotein (MbovP739)	MLRSAQLSLESLLKV	546-560	
	LRSAQLSLESLLKVE	547-561	
	NMLRSAQLSLESLLK	545-559	
	RSAQLSLESLLKVEK	548-562	
	ARTSGYIMPLASVVT	493-507	
	RTSGYIMPLASVVTE	494-508	
	IARTSGYIMPLASVV	492-506	
	TSQMSNTVMQAMIEQ	576-590	
	SQMSNTVMQAMIEQT	577-591	
	TSGYIMPLASVVTEA	495-509	
	KTSQMSNTVMQAMIE	575-589	
	QIFRFQSAISFAASV	378-392	H2-IAb, H2-IAd

SGYIMPLASVVTEAT	496-510	
LNMLRSAQLSLESLL	544-558	
WQIFRFQSAISFAAS	377-391	H2-IAb, H2-IAd
SWQIFRFQSAISFAA	376-390	
QMSNTVMQAMIEQTE	578-592	
IFRFQSAISFAASVG	379-393	H2-IAd, H2-IAb
KIFIKAGAILLPAAA	3-17	
FRFQSAISFAASVGA	380-394	H2-IAb, H2-IAd
DKTSQMSNTVMQAMI	574-588	
SAQLSLESLLKVEKG	549-563	
YGEYQLLKKVAHAIE	94-108	
EYGEYQLLKKVAHAI	93-107	
QSAISFAASVGAYQN	383-397	
SAISFAASVGAYQNK	384-398	
IKAGAILLPAAAGLS	6-20	

	KAGAILLPAAAGLSV	7-21		
	KLNMLRSAQLSLESL	543-557		
	KKIFIKAGAILLPAA	2-16		
	DDKTSQMSNTVMQAM	573-587		
Putative Lipoprotein (MbovP116)	TKLVMLSMPIALFAP	5-19	KLVMLSMPIALFAPV	6-20
	KTKLVMLSMPIALFA	4-18		
	KKTKLVMLSMPIALF	3-17		
	LVMLSMPIALFAPVL	7-21		
	VMLSMPIALFAPVLS	8-22		
	NKKTKLVMLSMPIAL	2-16		
	MNKKTKLVMLSMPIA	1-15		
	MLSMPIALFAPVLSA	9-23		
	NNLLSQVGVKISKDL	174-188		
Putative Lipoprotein (MbovP275)	MKKIKILSSIGILAP	1-15		
	KIKILSSIGILAPVL	3-17		

	IKILSSIGILAPVLA	4-18		
	ILAPVLAIPLVAARC	12-26		
Dihydrolipoamide dehydrogenase (DLD)	MHERKAKVVAKLSGG	149-163	AARSALSKLTAEKLN	518-532
	KMHERKAKVVAKLSG	148-162	EAARSALSKLTAEKL	517-531
	ARSALSKLTAEKLNE	519-533		
	VKMHERKAKVVAKLS	147-161		
	GGYLAAEMAGKAGLK	75-89		
	AQMYASMGTKVTIIQ	256-270		
	TNQNMLAHVAYIHAV	376-390		
	IELGARREVKVDKFL	348-362		
	GYLAAEMAGKAGLKT	76-90		
	FAQMYASMGTKVTII	255-269		
	PGGYLAAEMAGKAGL	74-88		
	HERKAKVVAKLSGGV	150-164		

LAAEMAGKAGLKTLI	78-92
YLAAEMAGKAGLKTL	77-91
LGARREVKVDKFLRT	350-364
QMYASMGTKVTIIQR	257-271
NQNMLAHVAYIHAVT	377-391
VTNQNMLAHVAYIHA	375-389
WEAARSALSKLTAEK	516-530
ELGARREVKVDKFLR	349-363
EFAQMYASMGTKVTI	254-268
GGVKFLMKASKVQTE	162-176
RSALSKLTAEKLNER	520-534
KFLMKASKVQTEEGV	165-179
GPGGYLAAEMAGKAG	73-87
GVKFLMKASKVQTEE	163-177
VKFLMKASKVQTEEG	164-178

ERKAKVVAKLSGGVK	151-165
GPNVTDYVAELALAI	478-492
MYASMGTKVTIIQRE	258-272
PNVTDYVAELALAIE	479-493
KNVILATGSHANRMK	198-212
NVTDYVAELALAIEK	480-494
GKNVILATGSHANRM	197-211
GIELGARREVKVDKF	347-361
VTDYVAELALAIEKR	481-495
WVKMHERKAKVVAKL	146-160
YKFATLGKAIAAEDT	443-457
IWEAARSALSKLTAE	515-529
IIWEAARSALSKLTA	514-528
VSKYKFATLGKAIAA	440-454
NVILATGSHANRMKF	199-213

	SKYKFATLGKAIAAE	441-455		
	VEFAQMYASMGTKVT	253-267		
XAA-Pro aminopeptidase (pepP)	ESFDEIVASGPNSAE	174-188	FDEIVASGPNSAEPH	176-190
	KESFDEIVASGPNSA	173-187	DEIVASGPNSAEPHH	177-191
	EIVASGPNSAEPHHH	178-192	SFDEIVASGPNSAEP	175-189
	IVASGPNSAEPHHHP	179-193		
	KSEEEIQAMQEVINI	123-137		
Transketolase (Tkt)	NINQKVVASMQAIAL	2-16	QKVVASMQAIALDSI	5-19
	VVASMQAIALDSINN	7-21	KVVASMQAIALDSIN	6-20
	ITHEALQIASVMKLN	161-175	NQKVVASMQAIALDS	4-18
	GITHEALQIASVMKL	160-174	INQKVVASMQAIALD	3-17
	EGITHEALQIASVMK	159-173	VASMQAIALDSINNA	8-22
	MNINQKVVASMQAIA	1-15		
	ASMQAIALDSINNAG	9-23		
	QEGITHEALQIASVM	158-172		

	LRLGGIMKLPAVHLY	431-445
	MGLNYAYNNTSTQVA	481-501
	RLGGIMKLPAVHLYS	432-446
	LQEGITHEALQIASV	157-171
	LGGIMKLPAVHLYSH	433-447
	GLNYAYNNTSTQVAI	488-502
Leucyl aminopeptidase (pepA)	KPSRSMVSMKFDMSG	243-257
	IVAATMKAIAQLKPK	260-274
	AIVAATMKAIAQLKP	259-273
	PSRSMVSMKFDMSGS	244-258
	LKPSRSMVSMKFDMS	242-256
	SRSMVSMKFDMSGSA	245-259
	VAATMKAIAQLKPKK	261-275
	AATMKAIAQLKPKKN	262-276
	MSGSAIVAATMKAIA	255-269

LNATRLIDVATLTGA	331-345
KVLNATRLIDVATLT	329-343
VLNATRLIDVATLTG	330-344
LEGFFKGLAVNANRN	61-75
DLEGFFKGLAVNANR	60-74
SDLEGFFKGLAVNAN	59-73

The predicted epitope is the result of consensus prediction between NN_align and SMM_align algorithm methods. Score is categorized according to IEDB (Immune Epitope Data Base) selection criteria based on a consensus percentile rank of the top 10 % (corresponding to 1000nM) as intermediate binding affinity (Percentile rank < 3 %) and high binding affinity (Percentile rank < 0.5 %).

Table S9: The 3D structure template and description used for epitope prediction

Protein	PDB ID	Description of 3D structure template molecule
Lipoprotein (MbovP579)	2WN4*	Structural Basis For Substrate Recognition In The Enzymatic Component Of Adp-Ribosyltransferase (Sundriyal et al., 2009)
Putative Lipoprotein (MbovP739)	4GL2*	Structural Basis For Dsrna Duplex Backbone Recognition By Mda5 (Wu et al., 2013)
Putative Lipoprotein (MbovP116)	2PNS*	1.9 Angstrom Resolution Crystal Structure Of A Plant Cysteine Protease Ervatamin-C Refinement With Cdna Derived Amino Acid Sequence
Putative Lipoprotein (MbovP275)	4U0Q*	Plasmodium Falciparum Reticulocyte-binding Protein Homologue 5 (pfrh5) Bound To Basigin
Dihydrolipoamide dehydrogenase (DLD)	1EBD	Dihydrolipoamide Dehydrogenase Complexed With The Binding Domain Of The Dihydrolipoamide Acetylase (Mande et al., 1996)
XAA-Pro aminopeptidase (pepP)	3Q6D	Xaa-Pro Dipeptidase From Bacillus Anthracis
Transketolase (Tkt)	3HYL	Crystal Structure of Transketolase From Bacillus Anthracis (Maltseva et al., To be published)

Leucyl aminopeptidase (pepA)	3JRU	Crystal Structure Of Leucyl Aminopeptidase (Pepa) From Xoo0834, Xanthomonas Oryzae Pv. Oryzae Kac (Natarajan et al., To be Published)

3D structure templates were modelled by both Ellipro and SWISS-MODEL Workspace.

* 3D structure template modelled only by Ellipro but not by SWISS-MODEL Workspace

Table S10: Conformational B cell epitope at minimum cut-off score of 0.8.

Protein- PDB ID	Residue	No.	Score
Lipoprotein (MbovP579)- 2WN4			
Epitope 1	$KEWERKE^{24\text{-}30}ER^{32\text{-}33}EQ^{35\text{-}36}$	11	0.916
Epitope 2	$SNGPVNNPNPE^{267\text{-}277}DS^{279\text{-}280}$	13	0.91
Epitope 3	$IRTENQNEISLEK^{113\text{-}125}R^{199}VIDGKH^{201\text{-}206}$	20	0.882
Epitope 4	$KLERS^{37\text{-}41}\text{-}KE^{43\text{-}44}K^{89}$	8	0.873
Epitope 5	$ND^{236\text{-}237}\text{-}SNKLTPNELA^{239\text{-}248}R^{291}$	13	0.871
Epitope 6	$NK^{321\text{-}322}E^{324}$	3	0.82
Epitope 7	$LTLTSP^{311\text{-}316}$	6	0.807
Putative lipoprotein (MbovP739)-4GL2			
Epitope 1	$V^{990}NNST^{993\text{-}996}$	5	0.924
Epitope 2	$LKNN^{466\text{-}469}LKK^{471\text{-}473}VIP^{478\text{-}480}$	10	0.9
Epitope 3	$MKPEE^{864\text{-}868}AHK^{870\text{-}872}$	8	0.889
Epitope 4	$PSLIT^{900\text{-}904}CSVLACSGED^{910\text{-}919}NMTPEFKELY^{930\text{-}939}FK^{991\text{-}}$	28	0.885

	$^{992}I^{1008}$		
Epitope 5	$NEKFA^{741-745}$	5	0.88
Epitope 6	$N^{706}M^{709}YT^{712-713}$	4	0.878
Epitope 7	$H^{921}FPNL^{1010-1013}$	5	0.873
Epitope 8	$KKKKASEPG^{349-357}$	9	0.869
Epitope 9	$EN^{682-683}KM^{685-686}RL^{689-690}ENP^{692-694}$	9	0.868
Epitope 10	$R^{595}LDE^{669-671}R^{674}T^{678}$	6	0.853
Epitope 11	$R^{560}Y^{564}K^{596}F^{675}$	4	0.828
Putative Lipoprotein (MbovP116)-2PNS			
Epitope 1	MNK^{1-3}	3	0.951
Epitope 2	YGY^{96-98}	3	0.944
Epitope 3	$YKYT^{324-327}$	4	0.941
Epitope 4	$KIENF^{230-234}$	5	0.938
Epitope 5	$VEYKLF^{101-106}$	6	0.906
Putative Lipoprotein (MbovP275)-4U0Q			

Epitope 1	KNN$^{353-355}$	3	0.998
Epitope 2	DGLTLTFVMNK$^{193-203}$	11	0.854
Epitope 3	NSIVTVNE$^{100-107}$DDVHSDEIDLSKVTENDKNHYLD^{226-} ^{248}NAKDSKISFIVHNQ$^{339-352}$	45	0.821
Epitope 4	RKSVIDFLNSEEK$^{43-55}$NSEQAKLLNSFLESNTLKVNEESLK$^{147-173}$ SQSSGKLTNEFKKKAEELN$^{280-299}$	57	0.819
Epitope 5	APWKNIGLNNEK$^{210-224}$	12	0.802
Dihydrolipoamide dehydrogenase (DLD) -1EBD			
Epitope 1	K^{241}T^{253}EANGETKT$^{255-262}$	10	0.913
Epitope 2	AE$^{243-244}$T^{251}D^{264}	4	0.882
Epitope 3	HSEEMGIKAENVTI$^{70-83}$	14	0.86
Epitope 4	EGID$^{371-374}$I^{376}RKEDG$^{403-407}$GMTAED$^{433-438}$ALTIHAHPT^{440-} ^{448}ME$^{454-455}$AEVAL$^{457-461}$	32	0.859
Epitope 5	AIETE$^{7-11}$LGQK$^{31-34}$VDANT$^{122-126}$-GDSAQT^{132-}	27	0.841

	^{137}T^{139}K^{141}G^{282}EQI$^{284-286}$N^{308}		
Epitope 6	R^{162}EREDG$^{245-249}$D^{266}	7	0.829
XAA-Pro aminopeptidase (pepP)- 3Q6D			
Epitope 1	M^{1}Q^{4}D^{7}IFAEKNIDC$^{9-17}$NV$^{31-32}$T^{34}DG$^{36-37}$EKDKY^{42-}<br>^{47}VDSRYIECEKNAKNVEVRLLAGKSLKE$^{50-77}$FDQKGYKKV^{79-}<br>^{87}K^{91}IVYDEFDRLVKLINPKTIA$^{94-112}$	78	0.843
Epitope 2	EK$^{143-144}$ISWIIPGMTE$^{146-155}$Q^{157}TK$^{160-161}$SGPN^{182-}<br>^{185}HHPTDRRIRDGE$^{191-202}$LGRQNVSDKPE$^{225-235}$EK^{237-}<br>^{238}E^{245}RL$^{248-249}$EAVKPGIKSD$^{252-262}$KI$^{265-266}$RDYIQNKGYGSYF^{268-}<br>^{280}SSHSDYILEE$^{299-308}$M^{310}IPGL$^{320-323}$TADG$^{335-338}$	91	0.821
Epitope 3	K^{140}HLKLYGA$^{164-171}$	8	0.813
Transketolase (Tkt)-3HYL			
Epitope 1	ASKKETADV$^{547-555}$KALAVDGVDA$^{572-581}$-ES$^{601-602}$-PKAVTK^{605-}<br>^{610}-GLEGD$^{627-631}$-G^{652}-TVEN$^{654-657}$-VRKVKEML$^{659-666}$	45	0.909
Epitope 2	RDAYNI$^{141-146}$-T^{307}AE$^{310-311}$NTMLGEYAQAYPELAN$^{313-328}$	25	0.887

Epitope 3	E^{469}GASAP$^{639-643}$K^{646}-EE$^{649-650}$	9	0.86
Epitope 4	EK$^{22-23}$-NS$^{25-26}$D^{87}KN$^{90-91}$TIGFGSPNKSGK$^{248-259}$A^{261}PLGVEETKLTKEA$^{266-278}$AWTAEQDFHVAEE$^{280-292}$E^{295}	47	0.859
Epitope 5	I^{189}GD$^{193-194}$NR$^{196-197}$	5	0.822
Leucyl aminopeptidase (pepA)-3JRU			
Epitope 1	DAPASAAV$^{10-17}$FADKTL$^{25-30}$QA$^{35-36}$DSASQGRLTALLARGDVAG$^{38-56}$LHDLPGVAAP$^{64-73}$GDAGK$^{81-85}$	50	0.873
Epitope 2	K^{336}P^{413}WDEYQGL$^{415-421}$,DSTFAD$^{423-428}$IGGRW$^{432-436}$	20	0.863
Epitope 3	T^{104}TATLGKKKVDET$^{143-154}$	13	0.816

(A)

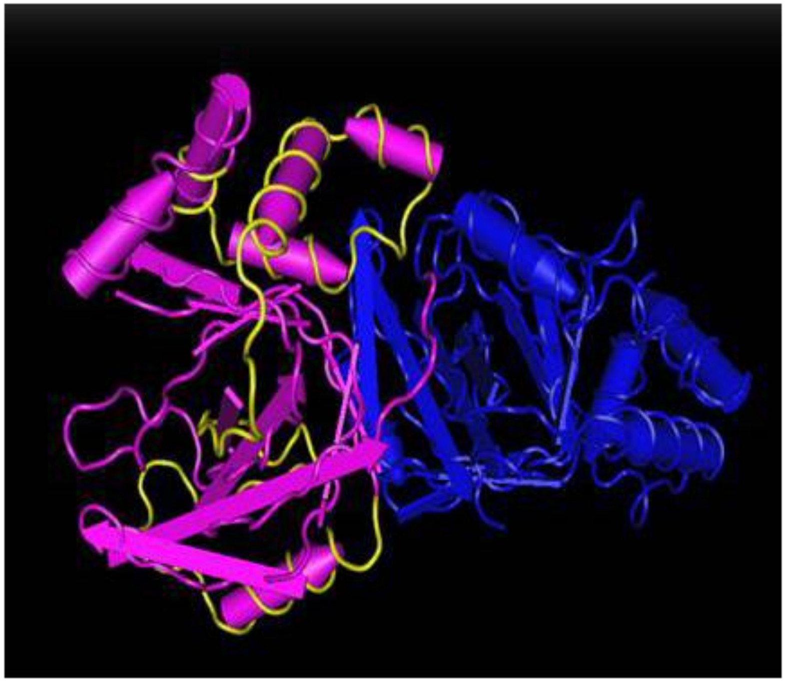

(B)

```
PDB ID-2WN4  89   LESYKKDSVEISKYSQTRNYFYDYQIEANSREKEYKELRNA--ISKNKIDKPMYVYYFES  146
                  L S +K  + +SKY +      D +   +SR+K YK++  A  IS    D   YV+
MbovP579     594  LTSLEKIDITLSKYDK------DMKKTKDSRDKPYKQVTPAQFIS----DAASYVF----  639

PDB ID-2WN4  147  PEKFAFNKVIRTENQNEISLEKFNEFKETIQNK  179
                  P K  F K   ++ +N+  +  +NE KE + NK
MbovP579     640  PVK-GFEKTDTSKIKNKYIVHTYNELKEAVTNK  671
```

FIG.S1. Three Dimensional (3D) Structure Template for MbovP579 modeled by Ellipro and sequence alignment. *(A),* worms style representation of the ADP-ribosyltransferase Toxin CDTa (PDB ID- 2WN4) from *Clostridium* difficile generated with Cn3D 4.3 software. The pink color represent N-terminal domain and the blue color represent C-terminal domain of CDTa, whereas the yellow color chain represent the aligned amino acids between CDTa and MbovP579 *(B),* NCBI database alignment between amino acid sequence of MbovP579 and CDTa. The aligned amino acids sequence is presented in red box.
